# From Preassociation to Chelation: A Survey of Cisplatin Interaction with Methionine at Molecular Level by IR Ion Spectroscopy and Computations

**DOI:** 10.1021/jasms.1c00152

**Published:** 2021-07-08

**Authors:** Roberto Paciotti, Davide Corinti, Philippe Maitre, Cecilia Coletti, Nazzareno Re, Barbara Chiavarino, Maria Elisa Crestoni, Simonetta Fornarini

**Affiliations:** † Dipartimento di Farmacia, Università G. D’Annunzio Chieti-Pescara, Via dei Vestini 31, Chieti I-66100, Italy; ‡ Dipartimento di Chimica e Tecnologie del Farmaco, Università di Roma “La Sapienza”, I-00185 Roma, Italy; § Institut de Chimie Physique, Université Paris-Saclay, CNRS, F-91405 Orsay, France

## Abstract

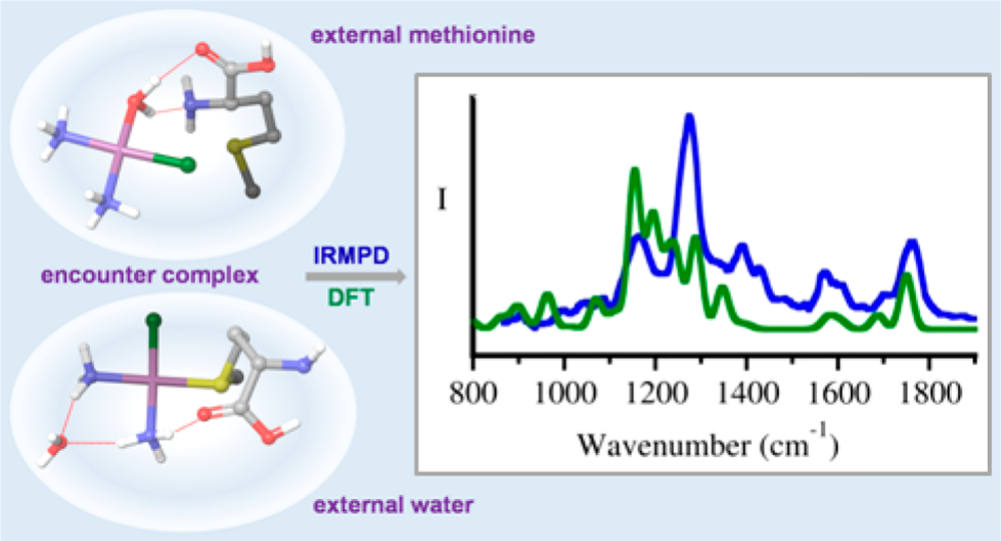

Methionine (Met) plays an important role in the metabolism of cisplatin anticancer drug. Yet, methionine platination in aqueous solution presents a highly complex pattern of interconnected paths and intermediates. This study reports on the reaction of methionine with the active aqua form of cisplatin, *cis*-[PtCl(NH_3_)_2_(H_2_O)]^+^, isolating the encounter complex of the reactant pair, {*cis*-[PtCl(NH_3_)_2_(H_2_O)]^+^·Met}, by electrospray ionization. In the unsolvated state, charged intermediates are characterized for their structure and photofragmentation behavior by IR ion spectroscopy combined with quantum-chemical calculations, obtaining an outline of the cisplatin–methionine reaction at a molecular level. To summarize the major findings: (i) the {*cis*-[PtCl(NH_3_)_2_(H_2_O)]^+^·Met} encounter complex, lying on the reaction coordinate of the Eigen-Wilkins preassociation mechanism for ligand substitution, is delivered in the gas phase and characterized by IR ion spectroscopy; (ii) upon vibrational excitation, ligand exchange occurs within {*cis*-[PtCl(NH_3_)_2_(H_2_O)]^+^·Met}, releasing water and *cis*-[PtCl(NH_3_)_2_(Met)]^+^, along the calculated energy profile; (iii) activated *cis*-[PtCl(NH_3_)_2_(Met)]^+^ ions undergo NH_3_ departure, forming a chelate complex, [PtCl(NH_3_)(Met)]^+^, whose structure is congruent with overwhelming S-Met ligation as the primary coordination step. The latter process involving ammonia loss marks a difference with the prevailing chloride replacement in protic solvent, pointing to the effect of a low-polarity environment.

## Introduction

Within the broad range of antineoplastic agents present in therapy, cisplatin (*cis-*diamminedichloroplatinum(II), *cis*-[PtCl_2_(NH_3_)_2_]) represents a landmark in medicinal inorganic chemistry, denoting a fine example of the therapeutic potential of complexes of exogenous metals. Its use in chemotherapy has been pivotal for over 40 years, and cisplatin is still considered a first-choice drug for several solid tumors, including head and neck and testicular cancers. In the cell environment, cisplatin undergoes hydrolysis, producing the corresponding aqua complex *cis*-[PtCl(NH_3_)_2_(H_2_O)]^+^, which is able to act as a platinating agent for DNA. Cisplatin reactivity is oriented preferentially toward the nucleophilic N7 of guanine, producing 1,2-intrastrand cross-links and thus inhibiting DNA transcription and replication.^1−6^ In spite of DNA being the main cisplatin target, platinum(II) is highly thiophilic and prone to interact with sulfur-donor ligands, which are mainly represented in the biological media by S-containing amino acids, i.e., cysteine and methionine, in peptides and proteins.^7−18^ It is worth noting that several proteins, not limited to serum transport systems and metal detoxification enzymes, are reported to be implicated in both the pharmacological and pharmacokinetic profiles of cisplatin.^19^ In order to improve our understanding of cisplatin interaction with peptides at a molecular level, in recent years we started to explore its binding motifs with proteinogenic amino acids, such as histidine^20,21^ and methionine,^21,22^ using electrospray ionization mass spectrometry (ESI-MS), high-resolution Fourier-transform ion cyclotron resonance (FT-ICR) mass spectrometry, and infrared multiple photon dissociation (IRMPD) spectroscopy combined with density functional theory (DFT) *ab initio* calculations. IRMPD spectroscopy is a powerful tool that allows one to reveal the vibrational features of mass-selected ions by monitoring the fragmentation produced by the absorption of photons that are resonant with the ion vibrational modes.^23−27^ Varying the photon energy allows us to obtain an IRMPD spectrum that can be compared with calculated IR spectra in order to gain structural information. It is worthy of note that the species is assayed in the gas phase, in the absence of solvent and associated band-broadening effects. IRMPD spectroscopy has been extensively used for the vibrational and structural characterization of transition metal complexes of both catalytic and biological relevance.^28−34^ In particular, cisplatin related platinum(II) complexes have been characterized.^34−36^ Previous reports have highlighted the versatility of ESI-MS together with the specificity of IRMPD spectroscopy in delivering detailed structural information on the monofunctional complexes *cis*-[PtCl(NH_3_)_2_(AA)]^+^, where AA is either Met or His.^20−22^ Furthermore, solutions of cisplatin and a simple ligand (L, chosen to model the nucleophilic functionalities of amino acids and nucleobases), assayed by ESI-MS and IRMPD spectroscopy have revealed the presence and role of the encounter complexes {*cis*-[PtCl(NH_3_)_2_(H_2_O)]^+^·L}.^37,38^ These species are formed by the diffusion controlled encounter of the aqua complex of cisplatin with the incoming ligand L and are the early actors in the substitution reaction of metal complexes in aqueous media, conforming to the Eigen–Wilkins reactant preassociation model.^39−42^ In the reported cases, the role of the encounter complex along the substitution reaction path of the cisplatin aqua complex with model ligands was assessed by observing the reaction to occur in the isolated species after collisional- or photoactivation, while IRMPD spectroscopy has afforded its vibrational and structural features. Moving one step further in assaying the cisplatin reaction with nucleophiles approaching the biomolecular targets, herein is reported the isolation and characterization of the adduct formally obtained by the preassociation of cisplatin aqua complex with methionine, [PtCl(NH_3_)_2_(H_2_O)(Met)]^+^. This species may be taken as a model for the early intermediate in the platination reaction of peptides presenting methionine residues.^39,43^ The [PtCl(NH_3_)_2_(H_2_O)(Met)]^+^ complex is isolated in the gas phase under long-lived conditions where it can be structurally assayed and activated. The reaction paths and intermediates are characterized by ESI-MS and IRMPD spectroscopy, backed by quantum chemical calculations. The dilute environment allows for fine control of the reaction occurring at the singly molecular level. This point may be significant in view of the multifaceted nature of the cisplatin reaction even with a simple molecule such as the amino acid methionine in aqueous solution. The reaction of cisplatin with methionine leads in fact to a variety of complexes where the amino acid performs as either mono- or bidentate-ligand, with a major end product being a *cis*-[Pt(NH_3_)_2_Met-(S,N)]^+/2+^ chelate.^44,45^ The reaction is dependent on pH because in strongly acidic conditions the *cis*-[Pt(NH_3_)_2_Met-(S,O)]^2+^ complex is preferentially formed, subsequently evolving to the more stable *cis*-[Pt(NH_3_)_2_Met-(S,N)]^2+^ at a higher pH.^46^ In the presence of 2 mol equiv of Met, diastereomeric *cis*-[Pt(Met-(S,N))_2_] bis-chelate complexes are observed.^44^

To summarize, an in depth understanding of the reactivity behavior of cisplatin with a simple amino acid target such as Met in an aqueous medium is highly difficult to achieve due to a complex pattern of interconnected paths and by the contribution of different hydrolyzed forms of cisplatin.^47,48^ Aiming to clarify the overall picture and to elucidate the role of the environment, the reaction of *cis*-[PtCl(NH_3_)_2_(H_2_O)]^+^, primarily formed in solution, has been examined starting from the preassociation adduct with the target Met reactant. The monosubstitution and bidentation processes and intermediates are characterized by both experimental and computational methods.

## Experimental Section

### Materials

Cisplatin and l-methionine from commercial sources were used as received. Stock aqueous solutions of l-methionine and of cisplatin were prepared at 10^–3^ M concentration, mixed in 1:1 molar ratio, and diluted with water to reach a final concentration of 5 × 10^–5^ M in each analyte in methanol/water (1:1 v/v). The solution of cisplatin was allowed to stand overnight prior to use.

### Mass Spectrometry and IRMPD Spectroscopy

Complexes of interest were obtained by ESI-MS of the solution prepared as described above. Typical ESI conditions were a flow rate of 120 μL/h, a capillary spray voltage set at −4.5 kV, nebulizer at 12 PSI, drying gas flow at 5 L/min, and drying gas temperature at 300 °C.

IRMPD spectroscopy experiments were performed using two instrumental setups, covering different ranges of the IR spectrum, namely, the IR fingerprint (900–1900 cm^–1^) and the X–H (X = C, N, O) stretching ranges (2900–3700 cm^–1^). The fingerprint range was explored using the beamline of the IR free electron laser (FEL) of the Centre Laser Infrarouge d’Orsay (CLIO). The electron energy of the FEL was set at 38 and 44 MeV in separate runs to optimize the laser power in the frequency ranges of interest. The IR beamline is coupled with a hybrid FT-ICR tandem mass spectrometer (APEX-Qe Bruker) equipped with a 7.0 T actively shielded magnet and coupled to a quadrupole–hexapole interface for mass-filtering and ion accumulation.^49^ The charged complexes of interest were mass selected in the quadrupole and collisionally cooled for 300 ms in the hexapole with argon as buffer gas, prior to IR irradiation. The isolated complexes were irradiated for 0.3–0.5 s with the IR FEL light, after which the resulting mass spectrum was recorded.

The X–H (X = C, N, O) bond stretching modes were investigated with a tunable KTP/KTA optical parametric oscillator/amplifier laser system (OPO/OPA, LaserVision) pumped by a non seeded Nd:YAG laser (Continuum Surelite II). The IR light is allowed to enter an ion trap mass spectrometer (Esquire 6000+, Bruker Daltonics), as described previously.^50^ In the trap, ions were mass-selected and accumulated for 30 ms prior to IR irradiation in the time scale of ca. 500 ms.

IR-FELs have been shown to be particularly well-suited for driving noncoherent IRMPD processes. The IR-FEL at CLIO provides ∼8 μs long trains of picosecond pulses delivered at 25 Hz. The high peak power (∼80 μJ per picosecond pulse) and the 16 ns time between two consecutive picosecond pulses are probably at the origin of the high IRMPD performance of IR-FELs. Conversely, the OPO/OPA laser used here delivers nanosecond pulses, which is determined by the Nd:YAG pulse length (∼5 ns). A typical pulse energy is 20–25 mJ, which is on the order of magnitude of an IR FEL macropulse energy (∼40 mJ). Lasers also differ by their bandwidth, which is controlled by the Nd:YAG pump (3–4 cm^–1^) in the case of the OPO/OPA but is much broader (typically Δλ/λ is 0.5%) in the case of CLIO IR-FEL. As a result, the IRMPD bandwidth observed in the 2900–3700 cm^–1^ range is smaller than that observed in the 900–1900 cm^–1^ region. Since electrosprayed ions are thermalized through multiple low-energy collisions at room temperature, it has been proposed^51^ that, while the IRMPD bandwidth is essentially controlled by ion temperature in the former range, it is controlled by the IR-FEL bandwidth in the latter.

IRMPD spectra are obtained by plotting the photofragmentation yield *R* (*R* = −ln[*I*_parent_/(I_parent_ + ∑*I*_fragment_)], where ln stands for natural logarithm and *I*_parent_ and *I*_fragment_ are the intensities of the precursor and fragment ions, respectively) as a function of the wavenumber.^52^

### Computational Methods

A conformational search was performed for both [PtCl(NH_3_)_2_(H_2_O)(Met)]^+^ and [PtCl(NH_3_)(Met)]^+^ complexes, using the Multiple Minimum Monte Carlo (MMMC) method with AMBER FF, implemented in Macromodel 9.6.^53^ For each isomer, similar conformations were grouped into clusters and their representative structures were minimized at the B3LYP/6-311+G(d,p) level of theory, using the pseudopotential LANL2DZ for platinum. Then, a visual inspection was performed in order to devise additional conformers that might have escaped from the conformational search.

Electronic energies, thermodynamic properties (zero point energy (ZPE), thermal corrections, and entropies), and harmonic frequencies were calculated by minimizing the selected structures at the B3LYP level of theory using a combined basis set, hereafter indicated as BS1, consisting of the 6-311+G(3df) basis set for the sulfur atom and 6-311+G(2df,pd) for the remaining atoms, except platinum, for which the pseudopotential LANL2TZ-f was adopted.

The relative energies of [PtCl(NH_3_)_2_(H_2_O)(Met)]^+^ were also computed at the ωB97X-D/BS1//B3LYP/BS1 level of theory to include the effect of long-range interactions in the energetics.

Harmonic frequencies computed for [PtCl(NH_3_)_2_(H_2_O)(Met)]^+^ and [PtCl(NH_3_)(Met)]^+^ complexes, in the 900–1900 and 2900–3700 cm^–1^ ranges, were scaled by 0.974 and 0.957, respectively.^37^

All the calculated spectra were convoluted assuming a Gaussian profile with an associated width (fwhm) of 15 cm^–1^ in the 900–1900 cm^–1^ range and 5 cm^–1^ in the 2900–3700 cm^–1^ frequency range. All quantum chemical calculations were performed using the Gaussian 09 package.^54^

## Results and Discussion

### Charged Intermediates in the Platination of Methionine

The aqua complex of cisplatin, *cis*-[PtCl(NH_3_)_2_(H_2_O)]^+^, obtained by the first reactive event involving hydrolysis of cisplatin in aqueous solution was found to yield the substitution product *cis*-[PtCl(NH_3_)_2_(Met)]^+^ when a 1:1 solution of cisplatin and Met in 50% aqueous methanol was examined by ESI-MS.^22^ Both reactant and product ions were thoroughly characterized by IRMPD spectroscopy, and the *cis*-[PtCl(NH_3_)_2_(Met)]^+^ complex was found to largely conform to a Met-S-ligated species.^22,55^ A preferential attack to the S-site of methionine is in accordance with the metal “softness”. Sampling by ESI was then able to intercept the transient charged complexes en route to the thermodynamically favored chelate complexes. It is recognized that ligand replacement occurs in solution by a multistep sequence outlined in eq 1 (Eigen–Wilkins reactant preassociation mechanism).^39^ The first step is a diffusion controlled entry of the incoming ligand L into the second coordination sphere of the metal ion complex. The rate-limiting event regards L/H_2_O (as in the present example) interchange between the two coordination spheres.
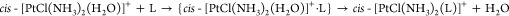
1

ESI-MS delivers in the gas phase and reveals the presence of ions formally corresponding to {*cis*-[PtCl(NH_3_)_2_(H_2_O)]^+^·L}, although MS alone does not allow us to discriminate from an isomeric {*cis*-[PtCl(NH_3_)_2_(L)]^+^·H_2_O} species or even from a less likely five-coordinate complex. Henceforth it is thus referred to as generically [PtCl(NH_3_)_2_(H_2_O)(L)]^+^.

**Figure 1:**
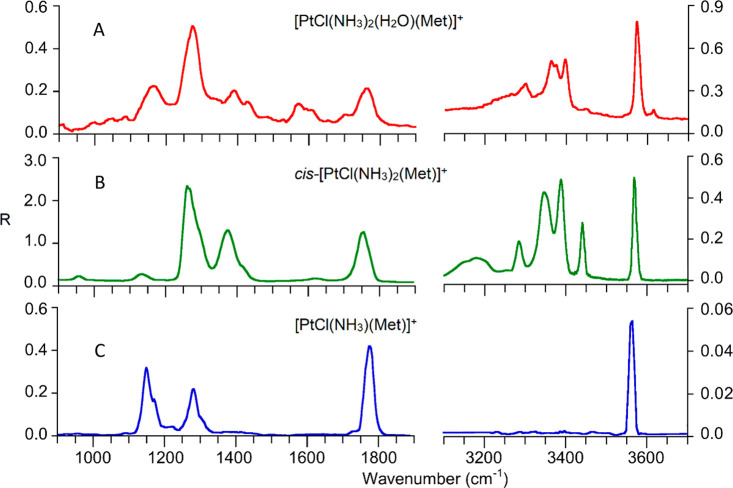
IRMPD spectra of [PtCl(NH_3_)_2_(H_2_O)(Met]^+^, *cis*-[PtCl(NH_3_)_2_(Met)]^+^, and [PtCl(NH_3_)(Met)]^+^.

The ESI mass spectrum of a cisplatin/Met solution (Figure S1 in the Supporting Information, SI) displays isotopic clusters congruent with *cis*-[PtCl(NH_3_)_2_(Met)]^+^ and [PtCl(NH_3_)_2_(H_2_O)(Met)]^+^ composition at *m*/*z* 412 and 430, respectively (where the isotopic cluster is represented by the *m*/*z* value of the first peak of conspicuous intensity, namely, the isotopic peak containing ^194^Pt and ^35^Cl). Both ions have been assayed by IRMPD spectroscopy in the 900–1900 cm^–1^ (IR fingerprint) and in the 3100–3700 cm^–1^ (X–H stretching, X = C, N, O) ranges. Figure 1A shows the IRMPD spectrum of [PtCl(NH_3_)_2_(H_2_O)(Met)]^+^. The photofragmentation process occurs by parallel routes involving a loss of either Met or H_2_O, with the latter process partly accompanied by a subsequent loss of NH_3_, as displayed in Figure S2. The dissociation of [PtCl(NH_3_)_2_(H_2_O)(Met)]^+^ ions (eq 2), following the absorption of multiple photons at an active vibrational frequency, largely favors a departure of H_2_O/H_2_O+NH_3_ over Met with a branching ratio equal to ca. 97:3. The presence of two competing dissociation channels may be ascribed to the presence of isomeric ions showing a distinct photofragmentation behavior. For example, the loss of Met could arise from a {*cis*-[PtCl(NH_3_)_2_(H_2_O)]^+^·Met} complex, holding Met in the outer coordination sphere, while H_2_O loss could reflect the external coordination of water, as in {*cis*-[PtCl(NH_3_)_2_(Met)]^+^·H_2_O}. However, this option is rather discarded in view of the IRMPD profile recorded along the two dissociation channels, as reported in Figure S3, which do not seem to present significant differences. In other words, both fragmentation routes are endured by the same species.
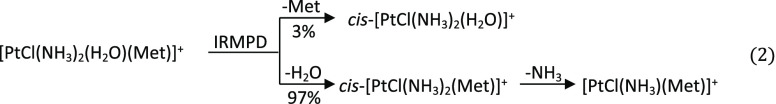
2

*cis*-[PtCl(NH_3_)_2_(Met)]^+^ ions, obtained by displacement by Met of the aqua ligand in *cis*-[PtCl(NH_3_)_2_(H_2_O)]^+^ ions, are characterized by the IRMPD spectrum depicted in Figure 1B, and their structure has been thoroughly elucidated in combination with quantum chemical calculations, also in comparison with isomeric *trans*-[PtCl(NH_3_)_2_(Met)]^+^ ions.^22^ The IRMPD process involves a loss of NH_3_, and the same cleavage occurs readily under collision induced dissociation, which has led to a proposal of the formation of a stable chelate complex.^22^ To gain structural information, the [PtCl(NH_3_)(Met)]^+^ complex, obtained by collision induced dissociation, was assayed by IRMPD spectroscopy (Figure 1C). This ion is less prone to undergo dissociation relative to its precursor. The photofragmentation mass spectrum reported in Figure S4 shows consecutive cleavages of NH_3_ and HCl followed by a further loss of NH_3_, with the latter fragment necessarily deriving from the amino acid backbone. It is indeed plausible that a stable chelate complex requires substantial activation to induce its fragmentation, and the reduced laser fluence in the 3100–3700 cm^–1^ range is likely responsible for apparently missing bands in the expected N–H stretch region (3250–3450 cm^–1^). The slowed rate of multiple photon absorption by less active oscillators may be counteracted by collisional deactivation. As a result, the photoframentation process that needs to reach a relatively high-energy threshold is inhibited. This finding is well--documented to affect IRMPD spectroscopy.^28,56^

While an analysis of the IRMPD spectra of *cis*-[PtCl(NH_3_)_2_(H_2_O)(Met)]^+^ and [PtCl(NH_3_)(Met)]^+^ is due, to elucidate ion structures, one may note common features in the three spectra displayed in Figure 1, in particular, a strong signal at ca. 3550 cm^–1^ (compatible with O—H stretching) and a band at ca. 1750 cm^–1^ (in the C=O stretch range), which suggests the presence of an unperturbed carboxylic group. Hence, the carbonyl group does not seem to be coordinated to the metal in any of the sampled species.

### Optimized Structures of [PtCl(NH_3_)(Met)]^+^

**Figure 2:**
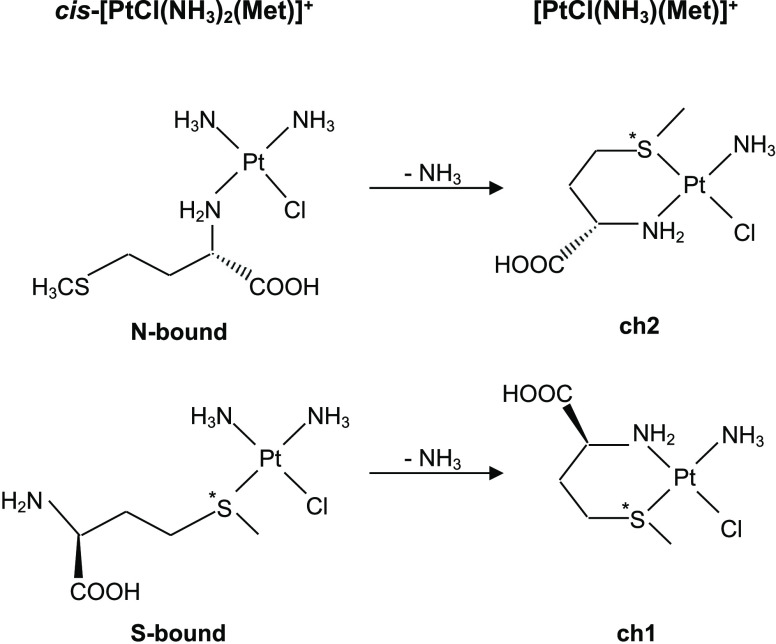
Formation of **ch1** and **ch2** isomers for [PtCl(NH_3_)(Met)]^+^ chelate complexes.

Reasoning on a chelate complex formation as the outcome of the second dissociation step in eq 1 (namely, *cis*-[PtCl(NH_3_)_2_(Met)]^+^ → [PtCl(NH_3_)(Met)]^+^ + NH_3_), the process may lead to **ch1** and **ch2** isomers depending on whether the precursor is an S- or N-ligated platinated methionine complex, respectively (Figure 2).

However, the monofunctional *cis*-[PtCl(NH_3_)_2_(Met)]^+^ ion was found to largely consist of an S-platinated complex,^22^ so that the resulting chelate complexes are expected to belong mainly to the **ch1** class. It is worthy of note that the observed *cis*-[PtCl(NH_3_)_2_(Met)]^+^ → [PtCl(NH_3_)(Met)]^+^ + NH_3_ process is a reactivity behavior specific of the unsolvated *cis*-[PtCl(NH_3_)_2_(Met)]^+^ complex. In protic solvent, ligand displacement preferentially involves chloride as the leaving group. Furthermore, isomers **ch1** and **ch2** can exist as pairs of epimers, **ch1_ss**/**ch1_sr** and **ch2_ss**/**ch2_sr**, where C_α_ and S atoms are both chiral centers. While C_α_ is always in *S* configuration (l-methionine), the S atom can be in either *R* or *S* configuration, which leads to different steric strain inferences.

**Figure 3:**
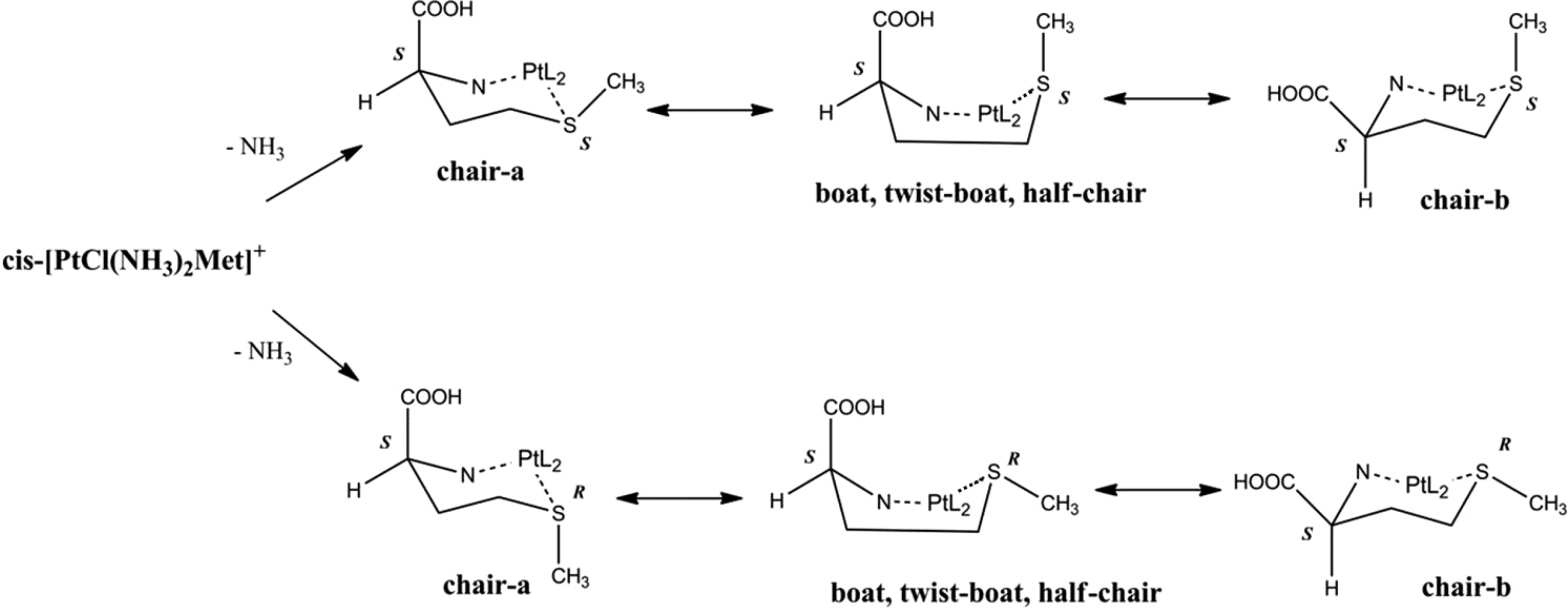
Accessible conformations of **ch1** and **ch2** isomers. Chair-a refers to a chair conformation where the C_α_ and S atoms are oriented upward and downward, respectively. Chair-b describes the opposite conformation. L_2_ stands for the NH_3_ and chlorido ligands bound to the Pt atom.

The structures include a six-membered ring, where three ring positions are occupied by N(Met), S, and Pt^2+^ atoms. The relevant conformations are summarized in Figure 3, and the two possible chair conformations are indicated as “a” and “b”, (e.g., **ch2_ss_b_1**). As mentioned earlier, while C_α_ has an *S* configuration coming from l-methionine, the sulfur atom depending on the chelation reaction can be either *S* or *R* (Figure 3), and for each configuration, it can bear an equatorial or axial methyl group.

**Figure 4:**
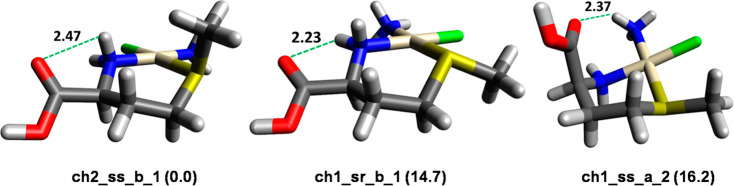
Lowest energy geometries for **ch1** and **ch2** isomers. Relative Gibbs energy values (kJ mol^–1^) are reported in parentheses. H-bond distances (Å) are represented by green dashed lines.

Figure 4 displays the lowest energy structures for **ch1** and **ch2** isomers. Their relative Gibbs energy (kJ mol^–1^) is reported in parentheses. An extended set of geometries lying in low-energy minima is shown in Figure S5. The lowest Gibbs energy structure is **ch2_ss_b-1**, characterized by a hydrogen bond linking C=O and NH_2_ groups of the amino acid ligand. The sulfur atom has *S* configuration, and consequently, the methyl group is in axial position.

On the contrary, the lowest energy structure in the set of conformers of the **ch1** isomer, **ch1_sr_b_1**, is characterized by CH_3_ in equatorial position with the Cl atom as the neighboring group, as shown in Figure 4. The corresponding epimer **ch1_ss_b_3** (Figure S5) is 5.0 kJ mol^–1^ higher in energy because of the repulsive interactions generated by the methyl group in axial position.

It is worth noting that **ch2** isomers are generally lower in energy than the corresponding **ch1** structures, indicating that the platinated complex is more stable when Cl, rather than NH_3_, is in a *trans* position to sulfur. **Ch2_ss_b_1** and **ch1_sr_b_1** isomers are characterized by the COOH group in equatorial position, whereas the **ch1_ss_a_2** isomer (Figure 4) is the lowest-energy structure where C=O can establish a hydrogen bond with NH_3_ rather than with NH_2_. This interaction is only possible for a **ch1** isomer with chair-a conformation holding COOH in the axial position.

### IRMPD Spectroscopy of [PtCl(NH_3_)(Met)]^+^

**Figure 5:**
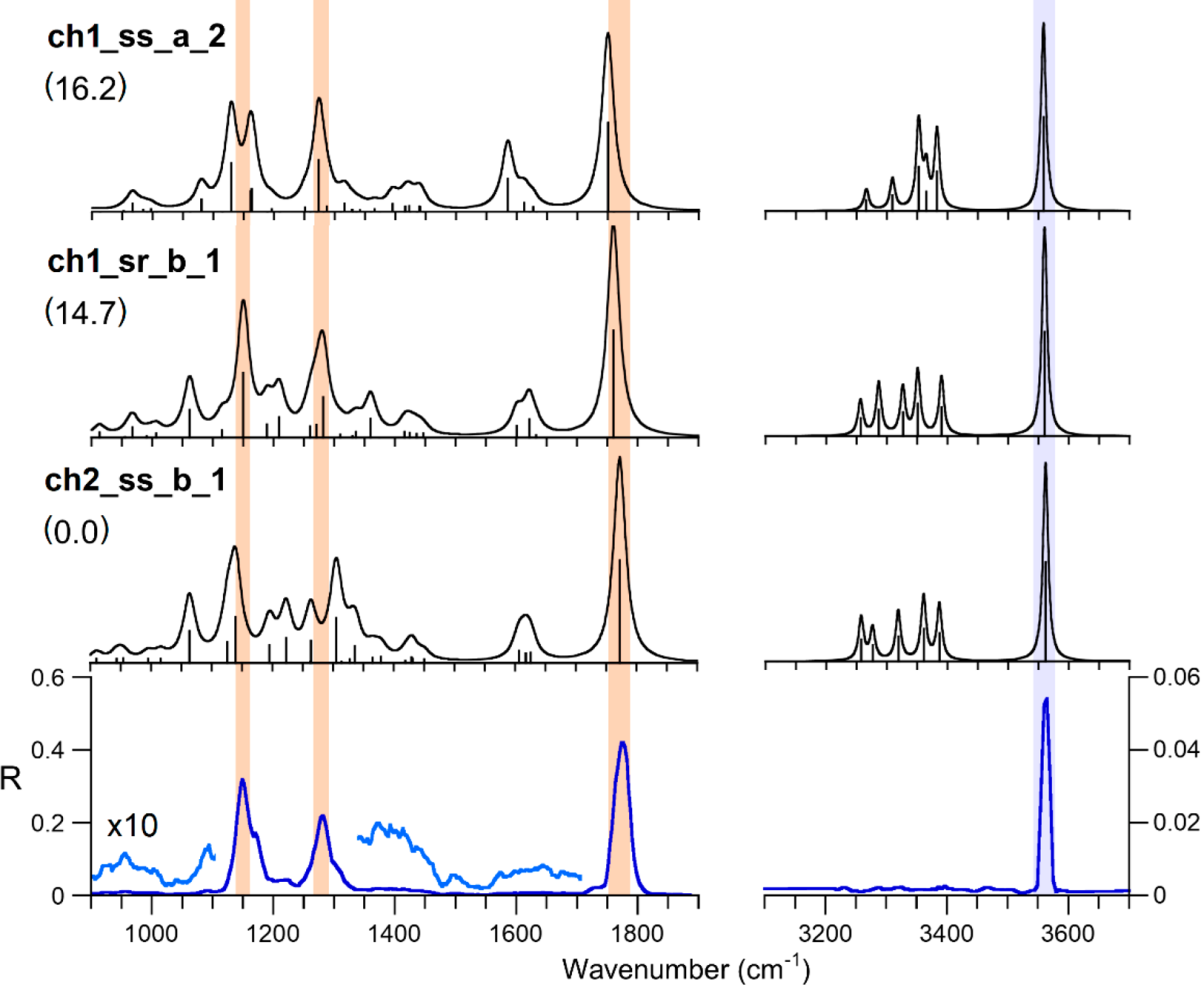
IRMPD spectrum of [PtCl(NH_3_)(Met)]^+^ (blue and pale blue profile) compared with the calculated IR spectra (black profiles) of the lowest lying conformers of the **ch2** and **ch1** families (**ch2_ss_b_1**, **ch1_sr_b_1**, and **ch1_ss_a_2**), computed at the B3LYP/BS1 level of theory. Theoretical frequencies have been scaled by 0.974 and 0.957 in the 900–1900 and 3100–3700 cm^–1^ ranges, respectively. Free energies relative to **ch2_ss_b_1** are reported in brackets (kJ mol^–1^).

The IRMPD spectrum of [PtCl(NH_3_)(Met)]^+^ generated by dissociation of *cis*-[PtCl(NH_3_)_2_(Met)]^+^ is reported in Figure 5 and compared with the calculated IR spectra of the low-energy structures of **ch1** and **ch2** isomers. The 3100–3700 cm^–1^ range presents a single absorption at 3563 cm^–1^, which can be accounted for by the OH stretching of the methionine carboxylic group. The fingerprint region (900–1900 cm^–1^) shows several features, with the most pronounced ones appearing at 1777, 1280, and 1148 cm^–1^.

In order to interpret the experimental bands and to characterize the structural features of the sampled ions, the IRMPD spectrum has been compared with calculated IR spectra of the low-energy structures of **ch1** and **ch2** isomers. Figure 5 shows the theoretical spectra of **ch2_ss_b_1**, the lowest-energy structure, and those of **ch1_sr_b_1** and **ch1_ss_a_2**, which are very close in energy (within 1.5 kJ mol^–1^). However, the relative Gibbs energy is not the only parameter to be considered to univocally assign the sampled gas-phase population to a certain isomer family. In fact, the two isomer families **ch1** and **ch2**, differing for the order of S- and N-platination, are generated starting from two different isomers of *cis*-[PtCl(NH_3_)(Met)]^+^ (Figure 1).

Therefore, the assayed gas-phase population of [PtCl(NH_3_)(Met)]^+^ is likely to reflect the isomeric distribution of the reactant complex, *cis-*[PtCl(NH_3_)_2_(Met)]^+^, which has already been experimentally assessed.^22^ In particular, methionine binding in the early monofunctional complex was found to involve almost exclusively the thioether function. Thus, the family of **ch1** conformers should be largely predominant in the chelate complex. This expectation is confirmed by experimental evidence. As a matter of fact, in spite of being very similar, the calculated spectra of the most stable conformers of **ch1** and **ch2** (such as **ch1_sr_b_1** and **ch2_ss_b_1**) show a few distinct differences in the low wavenumber range, which can guide the attribution of the assayed ion. The most notable difference is the NH_3_ umbrella mode, which is calculated at 1282 cm^–1^ for **ch1_sr_b_1**, in good agreement with the experiment, while in **ch2_ss_b_1** it is blue-shifted at 1305 cm^–1^. The experimental bands match fairly well with the calculated vibrational modes of **ch1_sr_b_1** and **ch1_ss_a_2**, which are very close in energy (1.5 kJ mol^–1^) and pertain to the same family. It is reasonable that the assayed ion population comprises both of them. The assignment of the vibrational modes is reported in Table S1 in the SI.

The experimental band at 3563 cm^–1^ is well-reproduced by both conformers (calculated absorptions at 3560 and 3558 cm^–1^ for **ch1_sr_b_1** and **ch1_ss_a_2**, respectively). In contrast, no features can be detected in the range around 3300 cm^–1^ where several vibrations are expected, comprising asymmetric and symmetric stretching modes of both the ammonia ligand and the amino group (Figure 3). As mentioned previously, the reason for the low fragmentation yield (to the limit of being undetectable) likely lies in the multiphotonic character of IRMPD, which hinders a proper detection of low intensity modes associated with a high-fragmentation threshold.^28,56^ In the fingerprint range, the most pronounced band at 1777 cm^–1^ is in agreement with the C=O stretching of **ch1_sr_b_1** and **ch1_ss_a_2**, calculated at 1760 and 1750 cm^–1^, respectively. Moving to lower wavenumbers, three experimental features are worth mentioning. In particular, (i) the intense band at 1280 cm^–1^, which has already been described and assigned to the NH_3_ umbrella mode of both **ch1** conformers; (ii) a small band at 1222 cm^–1^, which can be ascribed to the combination of NH_2_ and CH_2_ bending modes of **ch1_sr_b_1** (Table S1); and (iii) a band at 1148 cm^–1^ showing a shoulder at 1175 cm^–1^. The latter feature can be assigned to a combination of absorptions due to both **ch1_sr_b_1** and **ch1_ss_a_2** including OH bending mode coupled with NH_2_ wagging and CH_2_ twisting. While **ch1_sr_b_1** and **ch1_ss_a_2** stereoisomers account for the major IRMPD bands, the presence of other species contributing to the overall spectrum may not be discarded, as suggested for example by the tiny though meaningful shoulder on the red side of the C=O stretching absorption. The calculated IR spectra for the extended set of (stereo)isomers is reported in Figure S6.

It may thus be inferred that [PtCl(NH_3_)(Met)]^+^ ions formed in the gas phase by the dissociation of unsolvated *cis*-[PtCl(NH_3_)_2_(Met)]^+^ complexes display vibrational features consistent with a chelate complex derived from an S-Met ligated precursor. Diastereomeric forms (*SS* and *SR*) likely coexist, depending on the chirality of the coordinated thioether sulfur.

### Optimized Structures for [PtCl(NH_3_)_2_(H_2_O)(Met)]^+^

The optimized structures of *cis*-[PtCl(NH_3_)_2_(Met)]^+^ reported in previous work^22^ have provided the basis to obtain the starting geometries of the formal five-coordinate complexes, possibly responding to either {*cis*-[PtCl(NH_3_)_2_(H_2_O)]^+^·(Met)} or {*cis*-[PtCl(NH_3_)_2_Met]^+^·(H_2_O)}, indicated here as **ec1** and **ec2**, respectively. The former complex is an aqua platinum complex with a Met molecule in outer coordination, while in the second one, Met entered the first coordination sphere and water is noncovalently bound on the periphery.

**Figure 6:**
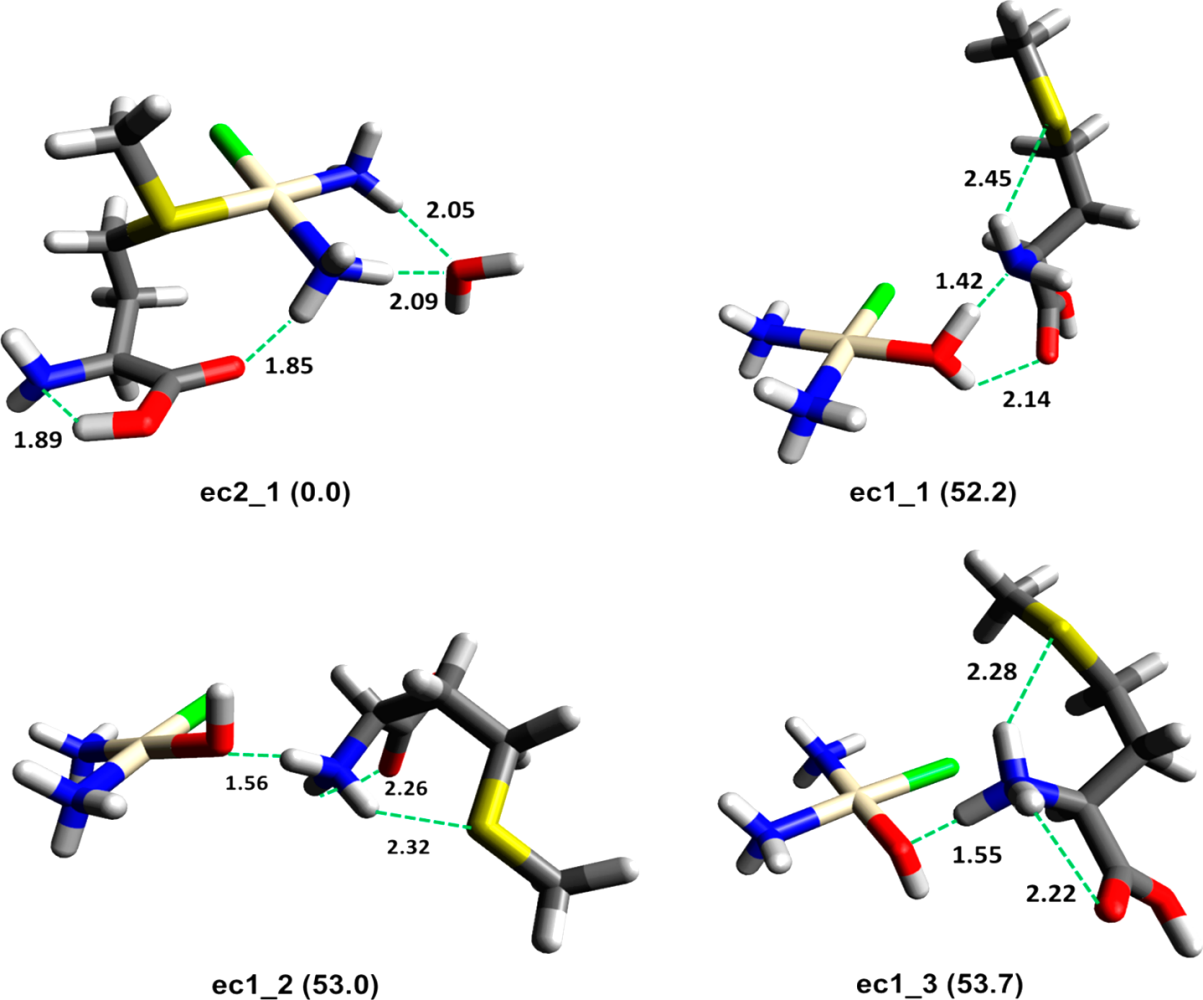
Lowest energy geometries for **ec1** and **ec2** isomers. Relative Gibbs energy values (kJ mol^–1^) are reported in parentheses. Hydrogen bond distances (Å) are indicated by green dashed lines.

As expected, the computational results have revealed an **ec2**-type ion to be thermodynamically favored, and the lowest energy structure is represented by **ec2_1**, where water is externally bound to *cis*-[PtCl(NH_3_)_2_(Met)]^+^ by means of two H bonds (Figure 6).

This arrangement (water is noncovalently bound on the periphery of a square planar platinum(II) complex) has been found to be the favored one also in other examples of [PtCl(NH_3_)_2_(L)(H_2_O)]^+^ complexes, where L is a simple ligand.^37,38^ In **ec2_1**, the COOH group is characterized by *anti*-geometry, allowing a hydrogen bond between the carboxylic H atom and the NH_2_ lone pair. A similar structure with COOH in *syn* conformation, **ec2_2**, is 5.5 kJ mol^–1^ higher in energy (Figure S7).

The lowest energy geometry of {*cis*-[PtCl(NH_3_)_2_(H_2_O)]^+^·Met}, where methionine is externally bound on the edge of the aqua complex, is **ec1_1**, placed at 52.2 kJ mol^–1^ Gibbs energy relative to **ec2_1** (Figure 6). In this structure, methionine interacts with *cis*-[PtCl(NH_3_)_2_(H_2_O)]^+^ by establishing two strong H-bonds with the water molecule, through the carbonyl oxygen and the nitrogen atoms. Moreover, an intramolecular hydrogen bond NH_2_···S is observed within methionine. Specifically, the H bond involving NH_2_ and the water H atom is significantly strong (*r* = 1.42 Å) with a consequent lengthening of the O–H bond (1.12 Å). These values suggest that such an intermolecular hydrogen bond (O–H···NH_2_) may represent an example of the so-called “hydrogen bridge”, where the H atom is shared and/or continuously transferred between two groups,^57^ leading to an equilibrium between {*cis*-[PtCl(NH_3_)_2_(H_2_O)]^+^·Met} and {*cis*-[PtCl(NH_3_)_2_(OH)]·MetH^+^}. It is found that the proton transfer can indeed occur, and the lowest energy structures of the corresponding isomers, **ec1_2** and **ec1_3**, are only 0.7 and 1.5 kJ mol^–1^ higher in energy, respectively. In the presence of a basic functional group the prototropic neutral form of the aqua complex of cisplatin is obtained within the encounter complex,^37^ as verified here. One can speculate that the adduct of cisplatin aqua complex and externally coordinated Met is in fact a dynamically equilibrating mixture of {*cis*-[PtCl(NH_3_)_2_(H_2_O)]^+^·(Met)} and {*cis*-[PtCl(NH_3_)_2_(OH)]·(MetH^+^)} ions, where the two partners interact by a strong hydrogen bridge.

Additional optimized structures for the **ec1** family of [PtCl(NH_3_)_2_(H_2_O)(Met)]^+^ complexes (Figure S8) lie at a relatively higher energy (>60 kJ mol^–1^ with respect to the reference **ec2_1**) and are not expected to contribute significantly to the sampled population. An extended set of optimized structures for both **ec1** and **ec2** families of isomers is displayed in Figures S7 and S8. In spite of specific search, no five-coordinate platinum complexes have been found lying in an energy minimum.

### IRMPD Spectroscopy of [PtCl(NH_3_)_2_(H_2_O)(Met)]^+^

**Figure 7:**
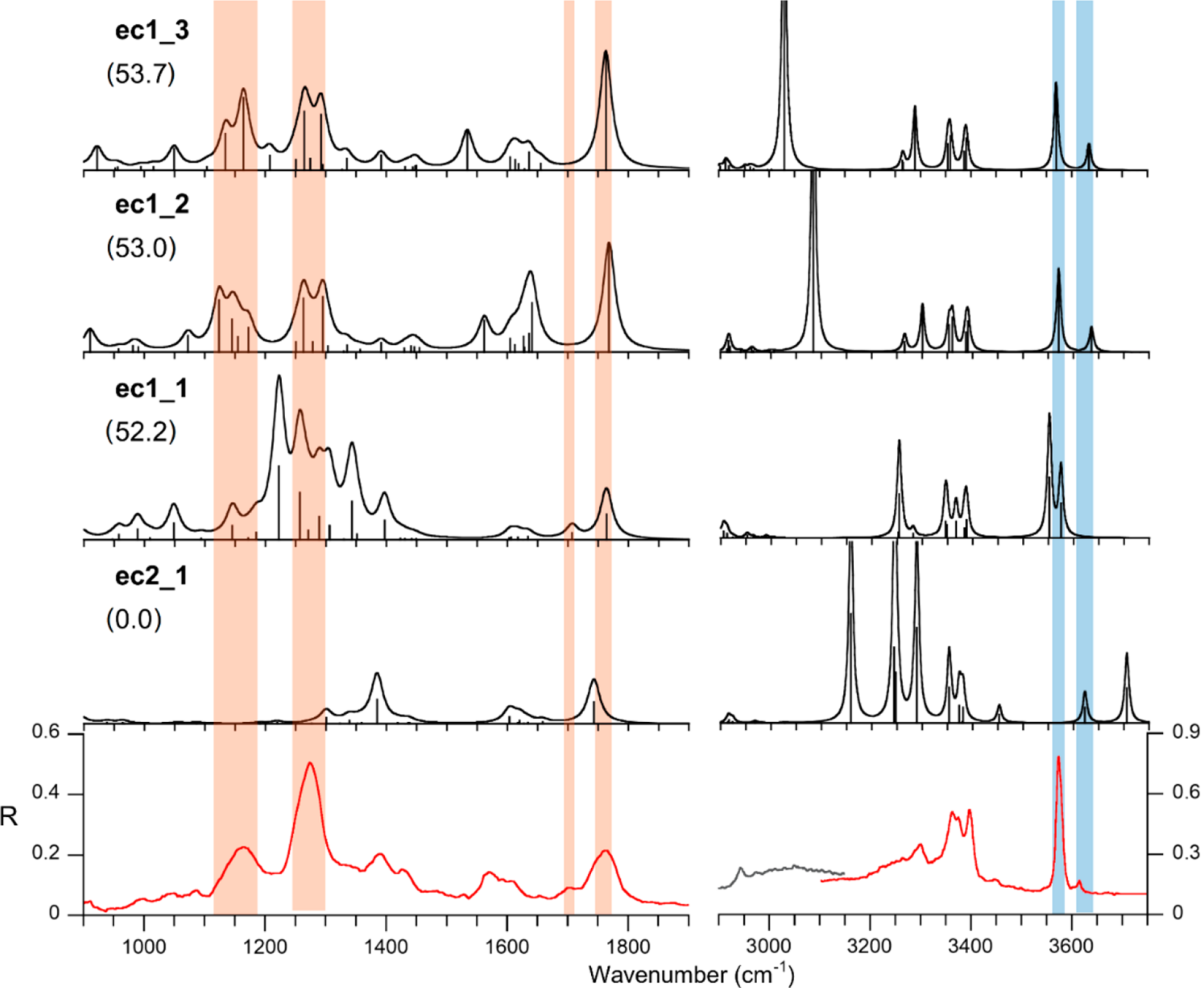
IRMPD spectrum of [PtCl(NH_3_)_2_(H_2_O)(Met)]^+^ (red and gray profile) compared with the calculated IR spectra (black profiles) of the lowest lying geometries of the **ec2** and **ec1** isomer families, computed at the B3LYP/BS1 level of theory. Theoretical frequencies have been scaled by 0.974 and 0.957 in the 900–1900 and the 3000–3700 cm^–1^ ranges, respectively. Free energies relative to **ec2_1** are reported in brackets (kJ mol^–1^).

The IRMPD spectrum of [PtCl(NH_3_)_2_(H_2_O)(Met)]^+^ ions was investigated in order to unveil the structure of sampled complex, aiming to discriminate between an encounter complex on the reactant, {*cis*-[PtCl(NH_3_)_2_(H_2_O)]^+^·(Met)}, or on the product, {*cis*-[PtCl(NH_3_)_2_(Met)]^+^·(H_2_O)}, side. To this end, the calculated IR spectra for the two isomer families **ec2** and **ec1** are examined against the experimental IRMPD spectrum. Figure 7 shows the IRMPD spectrum of [PtCl(NH_3_)_2_(H_2_O)(Met)]^+^ together with the calculated spectra of **ec2_1**, the lowest lying structure, and **ec1_1**, **ec1_2**, and **ec1_3**, the three most stable geometries of the **ec1** class. The large energy gap (52.2 kJ mol^–1^) between **ec2_1** and **ec1_1** is in agreement with the well-known bias for platinum binding to thio-containing nucleophiles.^9,17,18^ However, the relative thermodynamic stability may not predict which of the two isomer families is actually present. In fact, as already reported,^37,38^ the ESI process may not be able to deliver {*cis-*[PtCl(NH_3_)_2_(L)]^+^·(H_2_O)} like complexes in spite of them being generally lower in energy. The fragmentation pattern, privileging water loss, may reflect either the direct cleavage of noncovalently bound water (from an **ec2** isomer) or the ligand replacement event within vibrationally activated **ec1** ions, followed by leaving group departure. As already underlined, both photofragmentation paths arise from a single species or common mixture of isomers (Figure S3). Assaying [PtCl(NH_3_)_2_(H_2_O)(Met)]^+^ ions by IR spectroscopy is therefore mandatory to distinguish between **ec2** and **ec1** in order to comprehend the role of the sampled ion in the line of events related to the interaction of cisplatin with methionine.

Examining the spectra reported in Figure 7 allows us to rule out the presence of isomers belonging to the **ec2** family in the gas-phase population due to the absence in the experimental spectrum of any signal around 3700 cm^–1^. In this region, the calculated spectra of **ec2** species consistently show a pronounced band corresponding to the asymmetric stretching of water, as exemplified by the IR spectrum of **ec2_1** (Figure 7), where this mode is found at 3706 cm^–1^. This band is expected to be a signature of **ec2** ions, especially considering that these noncovalently bound complexes should be easily prone to photofragmentation. This point is in fact testified by the relatively high value of R, measuring the photofragmentation yield (Figure 7, Y scale). Computed IR spectra for the extended array of structures displayed in Figures S7 and S8 are shown in Figure S9. In the IRMPD spectrum, one may instead recognize IR signatures of **ec1_1**, **ec1_2**, and **ec1_3**, namely, the lowest lying structures of the **ec1** set that are likely contributing to the sampled ion population. Calculated vibrational modes are very similar for **ec1_2** and **ec1_3**, in line with their comparable structures characterized by common binding motifs so that the following discussion will only consider **ec1_1** and **ec1_2**. The assignment of vibrational modes to the experimental IRMPD bands is reported in Table S2. In the X–H (X = C, N, O) stretching range, two features appear above 3550 cm^–1^. The band at 3574 cm^–1^ can be assigned to the summed features of the OH stretching modes of water and of the carboxylic OH group of **ec1_1**, calculated at 3552 and 3575 cm^–1^, respectively. The same band is compatible with the carboxylic OH stretching of **ec1_2** at 3571 cm^–1^. The smaller signal at 3615 cm^–1^ can be assigned to the OH stretching of the hydroxo ligand in **ec1_2** (3636 cm^–1^). The cluster of signals between 3350 and 3400 cm^–1^ can be assigned to the asymmetric stretching of NH_3_ ligands in both **ec1_1** and **ec1_2** together with the asymmetric stretching of the β-amino group of methionine in **ec1_1**. The corresponding symmetric stretching is red-shifted due to the hydrogen bonding interaction with the S atom of the thioether group at 3255 cm^–1^ and accounts for the broad absorption around 3250 cm^–1^. The free NH stretching of the protonated β-amino group of **ec1_2** is, instead, calculated at 3301 cm^–1^, in agreement with the experimental signal at 3300 cm^–1^. The broad IRMPD activity that is observed in the 2950–3150 cm^–1^ span can be traced to the N—H stretching of the protonated amino group that is involved in H-bonding. Calculated harmonic IR spectra are known to misrepresent this kind of vibration due to their strong anharmonicity and to the multiphotonic character of the process.^58,59^ In the fingerprint range, the two bands at 1708 and 1766 cm^–1^ are well-simulated in the IR spectra of both **ec1_1** and **ec1_2**. In particular, the IR spectrum of **ec1_1** shows coupled vibrational modes, which include water scissoring and C=O stretching, which are calculated at 1707 and 1764 cm^–1^, respectively, while **ec1_2** presents the CO stretching mode at 1768 cm^–1^. The poorly resolved cluster of signals around 1600 cm^–1^ is in agreement with the calculated symmetric bending of the ammonia molecules and the amino group of both protomers. The intense bands between 1450 and 1150 cm^–1^ can arguably be attributed to a combination of the vibrational modes calculated in the same region for both **ec1_1** and **ec1_2**, which include NH_3_ umbrella modes, carboxylic OH bendings, and complex vibrational modes involving the amino acid backbone.

The interpretation of the IRMPD spectrum of [PtCl(NH_3_)_2_(H_2_O)(Met)]^+^ allowed by the analysis of the computational results thus points to an ion mixture comprising {*cis*-[PtCl(NH_3_)_2_(H_2_O)]^+^·(Met)} and {*cis*-[PtCl(NH_3_)_2_(HO)]·(MetH^+^)}. In any case, the noncovalent complex includes a cisplatin aqua complex associated with externally bound methionine. However, the most stable isomer is rather {*cis*-[PtCl(NH_3_)_2_(Met)]^+^·(H_2_O)}, and the question rises why this is not the observed species. An insight into this issue is provided by the energy profile for the ligand exchange reaction described in the following paragraph.

### Energy Profile for the Ligand Exchange Reaction in the Unsolvated Encounter Complex

**Figure 8:**
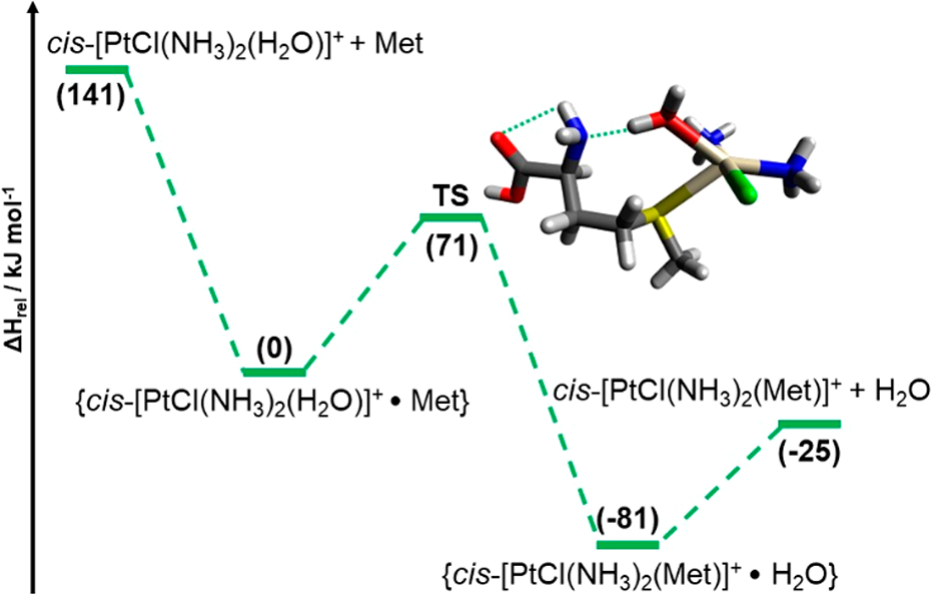
Energy profile for the reaction of *cis*-[PtCl(NH_3_)_2_(H_2_O)]^+^ with methionine. Relative enthalpy values at 298 K (in parentheses), computed at the ωB97X-D/BS1//B3LYP/BS1 level of theory, are reported in kJ mol^–1^. The geometry of the transition state (TS) is also shown.

In the calculated profile shown in Figure 8, relative enthalpy values are referred to the most stable geometry of the {*cis*-[PtCl(NH_3_)_2_(H_2_O)]^+^·(Met)} encounter complex of the reactant pair, namely, **ec1_1**. IRMPD spectroscopy has allowed us to clarify that this complex (existing also in prototropic forms **ec1_2–3**) is prone to undergo ligand displacement when activated by multiple photon absorption leading to the product {*cis*-[PtCl(NH_3_)_2_(Met)]^+^·(H_2_O)} complex (represented here by the lowest energy geometry of **ec2_1**) that promptly releases water. Thus, while recording the active vibrational frequencies, one also observes ligand exchange reactivity. The two encounter complexes are separated by an activation barrier of 71 kJ mol^–1^, corresponding to the relative enthalpy of the transition state depicted in Figure 8. As also shown in Figure S10, in the transition state, the elongation of the H_2_O–Pt bond (2.41 Å) is assisted by a H-bond involving the amino group of Met (1.71 Å). An incipient S–Pt bond (2.85 Å) is simultaneously formed with an overall five-coordinate geometry at the metal center. The angle formed by the entering S–Pt-leaving OH_2_ (S–Pt–O) is 76.1°, in agreement with a typical entering ligand–metal-leaving ligand angle (<90°).^60,61^ The Cartesian coordinates of the transition state are reported in Table S3. Note that, starting from the geometry of the encounter complex **ec1_1**, a reorientation of the Met side chain is needed for the sulfur atom to attack the metal center and for displacing the water molecule. IRC calculations were performed to verify that the transition state structure is indeed connected with ec1-type (reactant) and ec2-type (product) minima. The corresponding optimized geometries are given in Figure S11.

The energy profile may explain why the {*cis*-[PtCl(NH_3_)_2_(Met)]^+^·(H_2_O)} complex, in spite of being the most stable, is not in fact observed. The *cis*-[PtCl(NH_3_)_2_(Met)]^+^ ions, that are formed in solution and characterized in the gas-phase by IRMPD spectroscopy, are delivered by ESI due to progressive desolvation. However, the ESI conditions, promoting solvent departure, combined with the relatively low binding energy of water in this complex (amounting to 56 kJ mol^–1^, Figure 8) do not allow for the survival and isolation of {*cis*-[PtCl(NH_3_)_2_(Met)]^+^·(H_2_O)}. In contrast, the {*cis*-[PtCl(NH_3_)_2_(H_2_O)]^+^·(Met)} encounter complex lies in a fairly deep well relative to dissociation of the two partners (requiring 141 kJ mol^–1^) and it may thus be observed. The ligand substitution reaction in solution is clearly not fast enough to turn all cisplatin aqua complexes in the monofunctional Met derivative. Once delivered in the gas phase and activated by multiple photon absorption (or else by collision induced dissociation), the {*cis*-[PtCl(NH_3_)_2_(H_2_O)]^+^·(Met)} encounter complex undergoes ligand substitution, finally releasing water. The end products are lower in energy than the transition state for ligand displacement so that all complexes passing the activation barrier proceed to freely dissociate. At the same time, the high threshold energy for Met dissociation explains the highly unbalanced photofragmentation ratio for Met versus H_2_O departure, which is consistent with the behavior of comparable systems previously reported.^37,38^ Thus, the energy profile provides a neat account for the reaction occurring in the naked encounter complex of the two partners as retrieved from solution.

## Conclusions

As stated in the Introduction, the reaction of cisplatin with methionine in a biological context or even in aqueous solution is a complex issue due to the presence of different hydrolyzed forms of cisplatin and to an intricate reactivity network. In the present contribution, these known problems are circumvented, operating in the dilute gaseous environment and exploiting the ion selection, storage, and sampling ability of mass spectrometry-based methods. In this way it becomes possible to follow the progress of key steps in the ligand exchange reaction starting from the cisplatin aqua complex in the encounter complex with methionine, proceeding with aqua ligand exchange activated by the absorption of multiple IR photons and ending with chelate complex formation by NH_3_ departure (eq 3). Also this second ligand substitution step may be activated by the absorption of vibrational energy.
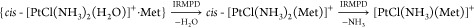
3

The structure of the assayed intermediates has been obtained by IRMPD spectroscopy aided by the analysis of computed IR spectra for candidate structures. By these means, the ion at *m*/*z* 430 of formal [PtCl(NH_3_)_2_(H_2_O)(Met)]^+^ composition has been unequivocally assigned to the {*cis*-[PtCl(NH_3_)_2_(H_2_O)]^+^·(Met)} complex (rather than to the {*cis*-[PtCl(NH_3_)_2_(Met)]^+^·(H_2_O)} isomer) and the [PtCl(NH_3_)(Met)]^+^ ion to a chelate complex deriving from the S-Met ligated *cis*-[PtCl(NH_3_)_2_(Met)]^+^ intermediate. Vibrational excitation of the {*cis*-[PtCl(NH_3_)_2_(H_2_O)]^+^·(Met)} encounter complexes is found to activate the H_2_O/Met ligand exchange process rather than dissociation into the free reactant pair, *cis*-[PtCl(NH_3_)_2_(H_2_O)]^+^·Met. This finding is well-accounted for by the computed energy profile. Finally, NH_3_ loss in the formation of the [PtCl(NH_3_)(Met)]^+^ chelate complex indicates that a low-polarity medium may significantly affect the process of leaving group departure.

## Supporting Information Available

Figures of mass spectra, IRMPD profiles, calculated IR spectra and optimized geometries and tables of IRMPD absorptions and calculated vibrational frequencies and Cartesian coordinates (PDF)
