# Supplementary material for: From Preassociation to Chelation: A Survey of Cisplatin Interaction with Methionine at Molecular Level by IR Ion Spectroscopy and Computations
Source: J Am Soc Mass Spectrom. 2021 Jul 8;32(8):2206–17. doi: 10.1021/jasms.1c00152 (PMC8397306; doi:10.1021/jasms.1c00152)
Supplement: Supplementary file 1 — js1c00152_si_001.pdf [file js1c00152_si_001.pdf]

Supporting Information for

From preassociation to chelation: a survey of cisplatin interaction with methionine at molecular level by IR ion spectroscopy and computations

Roberto Paciotti,[1‡] Davide Corinti,[2‡] Philippe Maitre,[3] Cecilia Coletti,[1] Nazzareno Re,[1] Barbara Chiavarino,[2] Maria Elisa Crestoni,[2] and Simonetta Fornarini[2]*

[1] Dipartimento di Farmacia, Università G. D'Annunzio Chieti-Pescara, Via dei Vestini 31, Chieti I-66100, Italy
[2] Dipartimento di Chimica e Tecnologie del Farmaco, Università di Roma "La Sapienza", I-00185 Roma, Italy
[3] Institut de Chimie Physique, Université Paris-Saclay, CNRS, F-91405 Orsay, France

Corresponding author's e-mail : simonetta.fornarini@uniroma1.it

Table of contents

p. S2 Figure S1. Mass spectrum of a solution of cisplatin and methionine (1:1) 5×10^{-5} M in methanol/water (50/50, v/v) recorded with an Esquire 6000 ion trap mass spectrometer.
p. S2 Figure S2. Mass spectrum recorded upon selection and irradiation at 1273 cm^{-1} of *cis*-[PtCl$(NH_3)_2(H_2O)$(Met)]$^+$ ions at *m/z* 430.
p. S3 Figure S3. IRMPD profiles of the primary fragmentation channels from [PtCl$(NH_3)_2(H_2O)$(Met)]$^+$ involving cleavage of either neutral methionine or H_2O.
p. S4 Figure S4. Mass spectrum recorded upon selection and irradiation at 1150 cm^{-1} of [PtCl(NH_3)(Met)]$^+$ ions at *m/z* 395.
p. S5 Figure S5. Optimized geometries for **ch1** and **ch2** isomers, [PtCl(NH_3)Met]$^+$.
p. S9 Figure S6. IRMPD spectrum of [PtCl(NH_3)(Met)]$^+$ (blue profile) compared with the calculated IR spectra (black profiles) of the lowest lying conformers of the **ch1** and **ch2** families, computed at B3LYP/BS1 level of theory.
p. S14 Figure S7. Optimized geometries for **ec2** isomers, {cis-[PtCl$(NH_3)_2$Met]$^+$ • H_2O}.
p. S15 Figure S8. Optimized geometries for **ec1** isomers, {cis-[PtCl$(NH_3)_2(H_2O)$]$^+$ • Met} and {cis-[PtCl$(NH_3)_2$(OH)]• MetH$^+$}.
p. S19 Figure S9. IRMPD spectrum of [PtCl$(NH_3)_2(H_2O)$(Met)]$^+$ (red profile) compared with the calculated IR spectra (black profiles) of the lowest lying geometries of the **ec2** and **ec1** isomer families, computed at B3LYP/BS1 level of theory.
p. S24 Figure S10. structure of transition state participating in the reaction pathway for the aqua ligand substitution reaction of cis-[PtCl$(NH_3)_2(H_2O)$]$^+$ with Met.
p. S24 Figure S11. Optimized geometries of the reactant (A) and of the product (B) obtained by intrinsic reaction coordinate (IRC) calculations.
p. S25 Table S1. IRMPD absorptions of [PtCl(NH_3)(Met)]$^+$ ions and calculated vibrational frequencies (cm^{-1}) for **ch1_sr_b_1** and **ch1_ss_a_2**.
p. S27 Table S2. IRMPD absorptions of [PtCl$(NH_3)_2(H_2O)$(Met)]$^+$ ions and calculated vibrational frequencies (cm^{-1}) for **ec1_1 and ec1_2**.
p. S25 Table S3. Cartesian coordinates of transition state computed for the aqua ligand substitution reaction of cis-[PtCl$(NH_3)_2(H_2O)$]$^+$ with Met

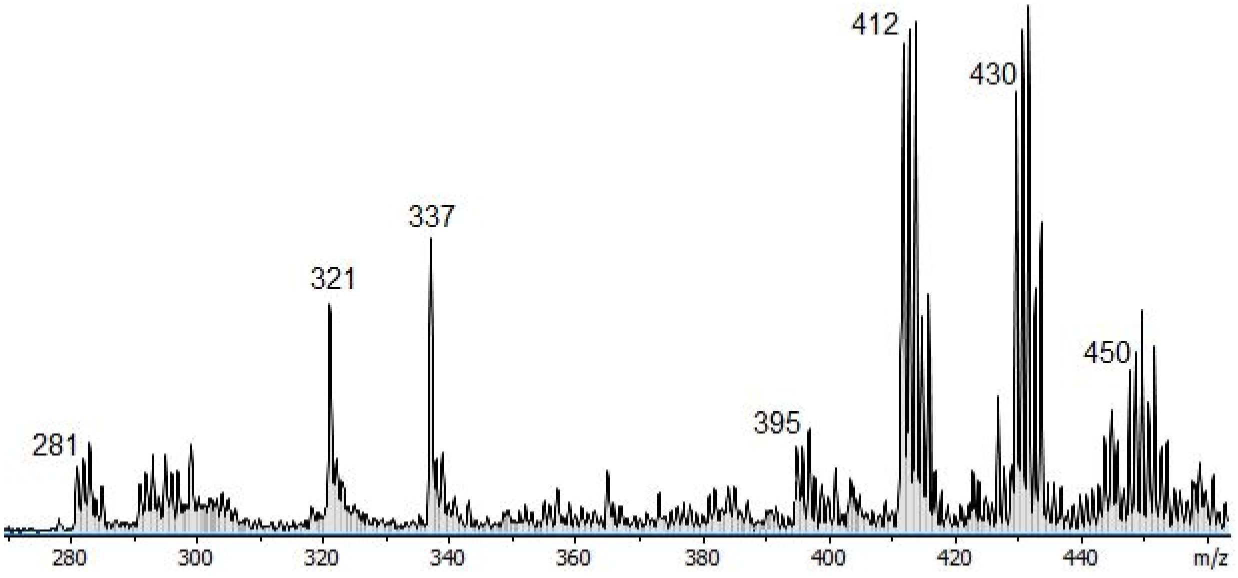

Figure S1. Mass spectrum of a solution of cisplatin and methionine (1:1) 5×10^{-5} M in methanol/water (50/50, v/v) recorded with an Esquire 6000 ion trap mass spectrometer. The cluster at *m/z* 412-416 corresponds to *cis*-$[PtCl(NH_3)_2(Met)]^+$, and the one at *m/z* 430-414 to the formal five-ligand complex $[PtCl(NH_3)_2Met(H_2O)]^+$. The signals at *m/z* 321, 337 are related to Met, i.e. $[Met_2+Na]^+$ and $[Met_2+K]^+$, respectively. *m/z* 281-285 is the *cis*-$[PtCl(NH_3)_2(H_2O)]^+$ complex.

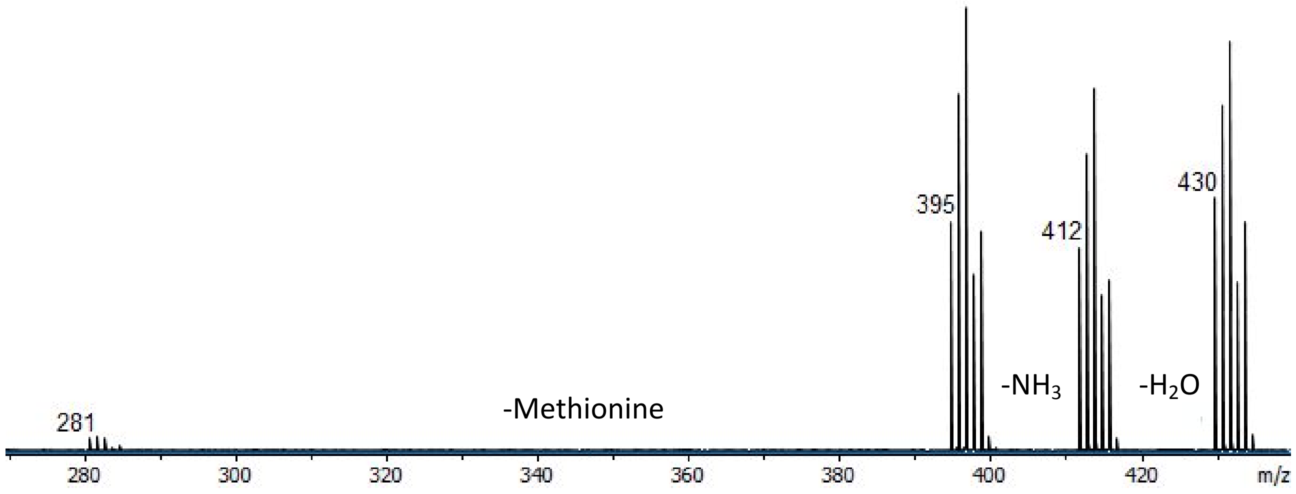

Figure S2. Mass spectrum recorded upon selection and irradiation at 1273 cm^{-1} of *cis*-$[PtCl(NH_3)_2(H_2O)(Met)]^+$ ions at *m/z* 430.

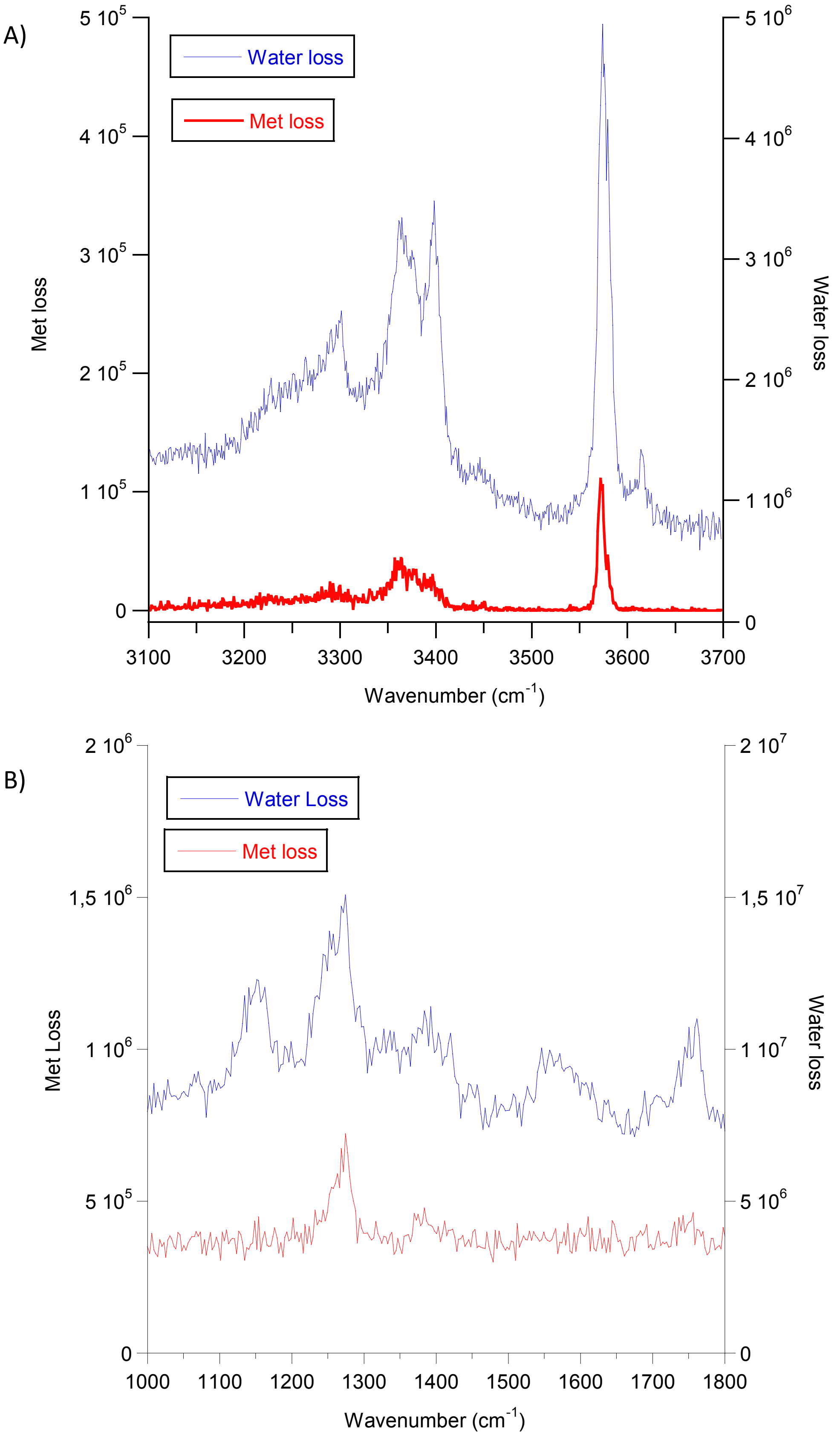

Figure S3. IRMPD profiles of the primary fragmentation channels from $[PtCl(NH_3)_2(H_2O)(Met)]^+$ involving cleavage of either neutral methionine (red) or H_2O (blue) in A) the XH stretching and B) fingerprint regions.

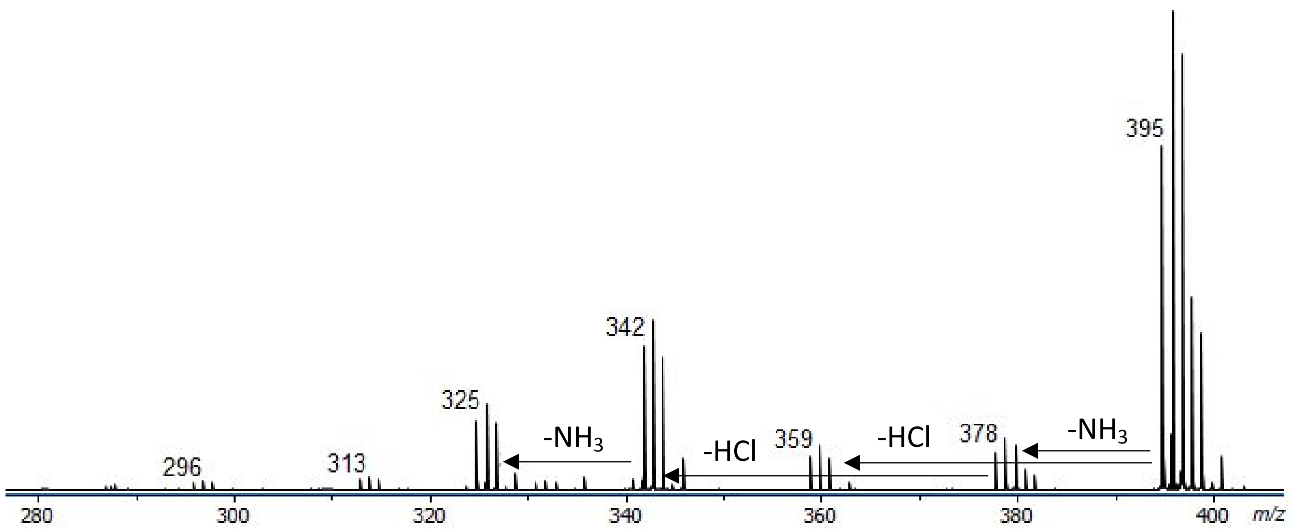

Figure S4. Mass spectrum recorded upon selection and irradiation at 1150 cm^{-1} of $[PtCl(NH_3)(Met)]^+$ ions at *m/z* 395.

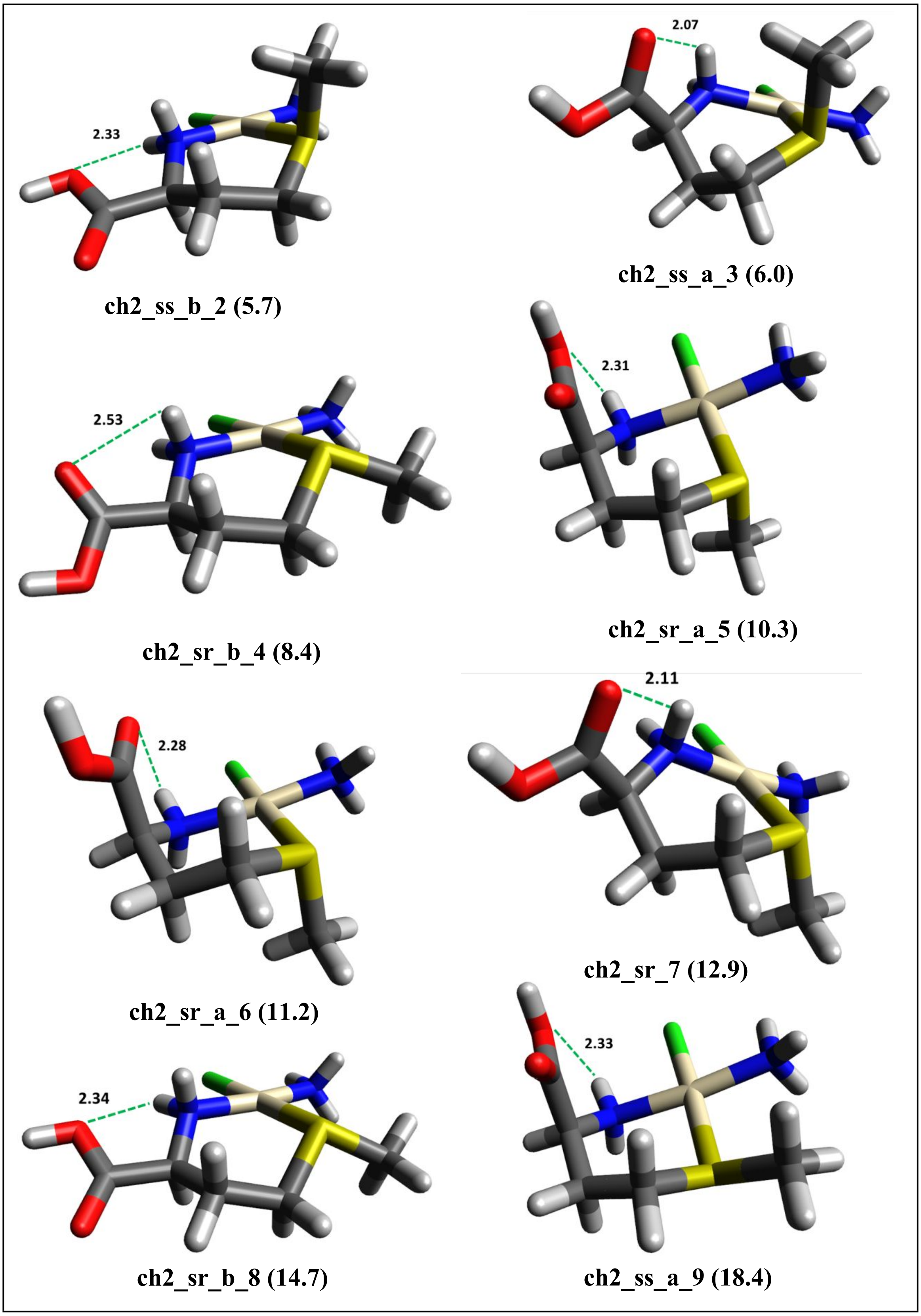

Figure S5. Optimized geometries for **ch1** and **ch2** isomers, $[PtCl(NH_3)Met]^+$. Relative Gibbs energy values (kJ mol^{-1}) are reported in parenthesis. Intramolecular hydrogen bond distances (Å) are represented by green dashed lines.

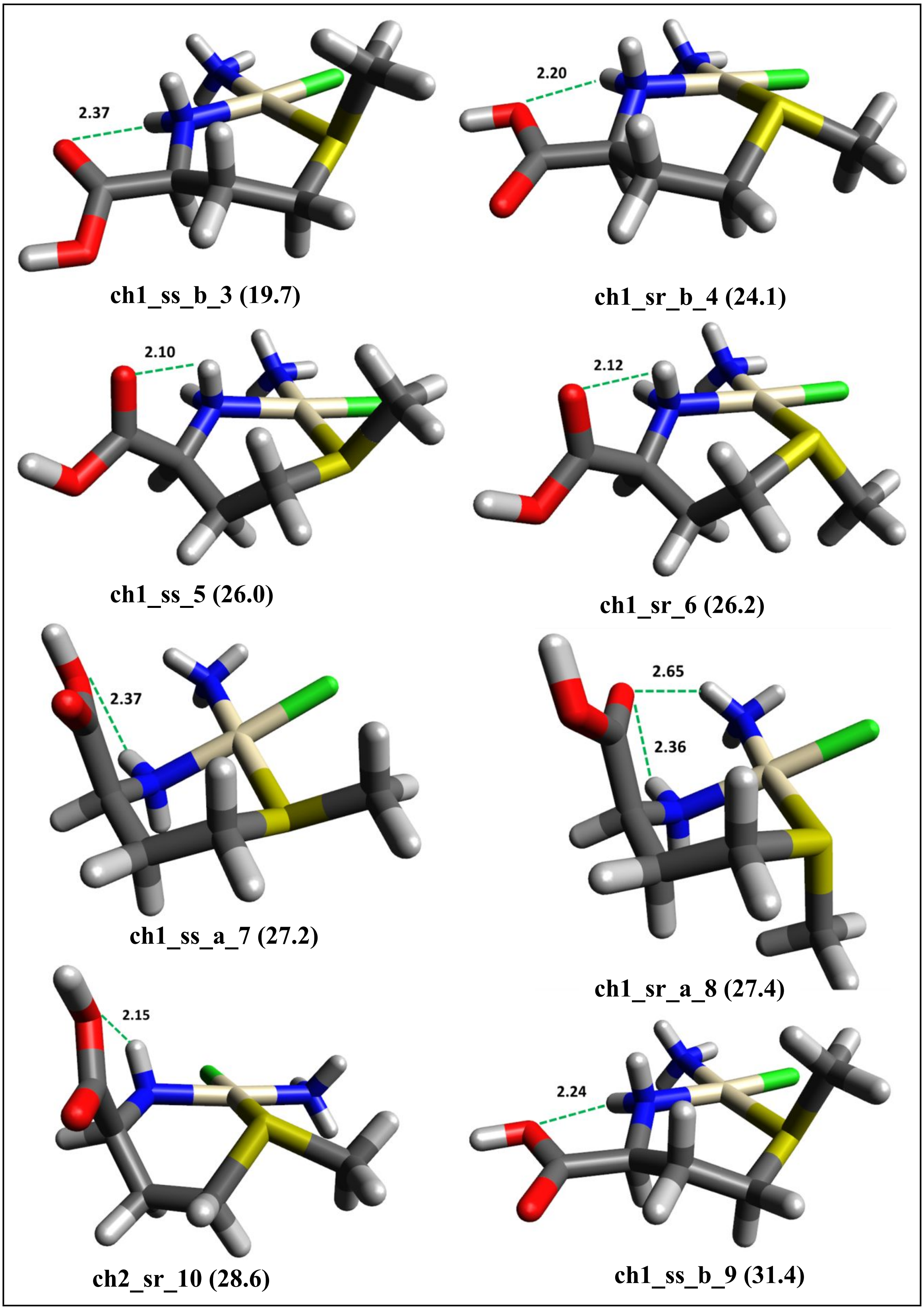

Figure S5 (continuation). Optimized geometries for **ch1** and **ch2** isomers, $[PtCl(NH_3)Met]^+$. Relative Gibbs energy values (kJ mol^{-1}) are reported in parenthesis. Intramolecular hydrogen bond distances (Å) are represented by green dashed lines.

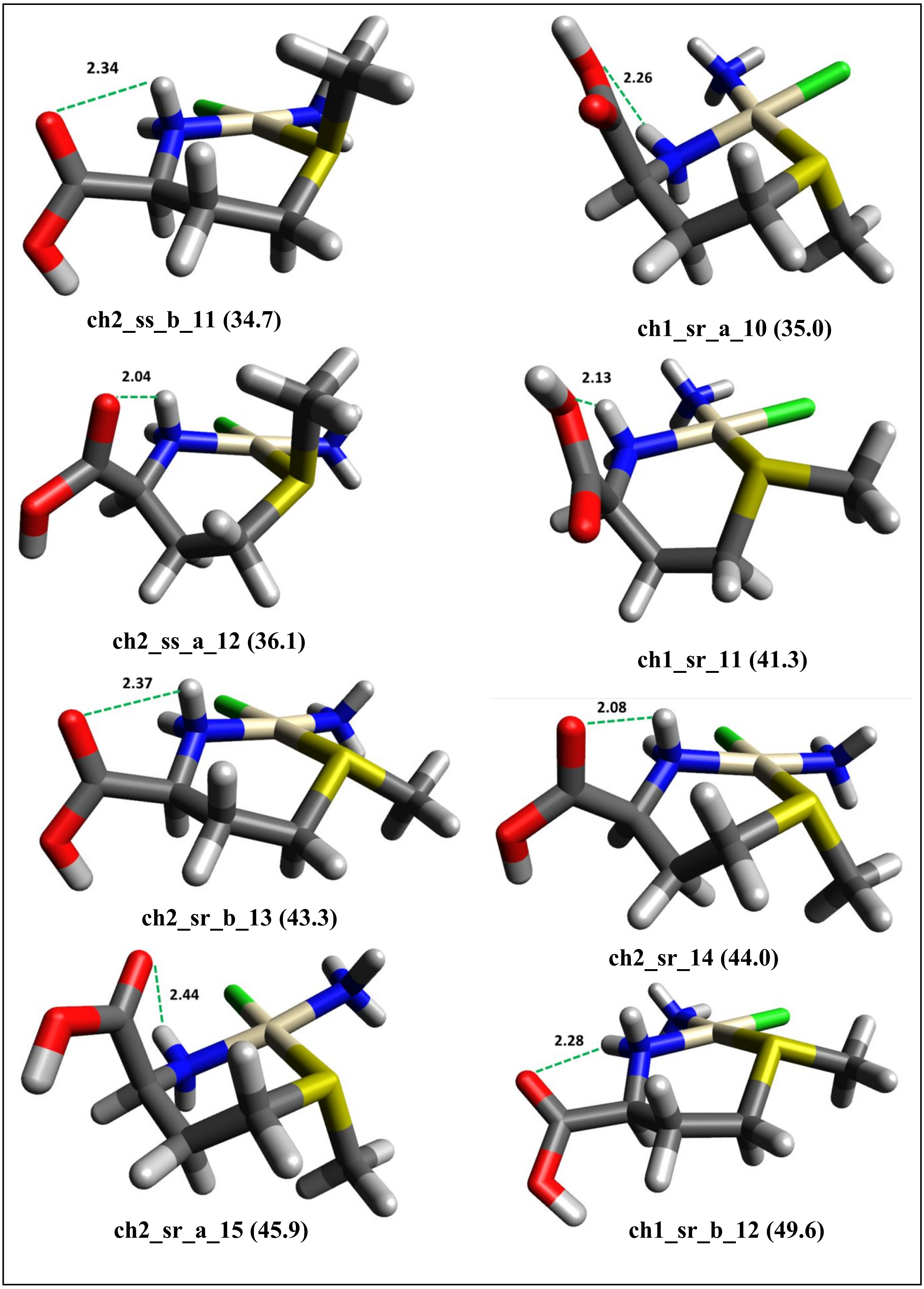

Figure S5 (continuation). Optimized geometries for **ch1** and **ch2** isomers, $[PtCl(NH_3)Met]^+$. Relative Gibbs energy values (kJ mol^{-1}) are reported in parenthesis. Intramolecular hydrogen bond distances (Å) are represented by green dashed lines.

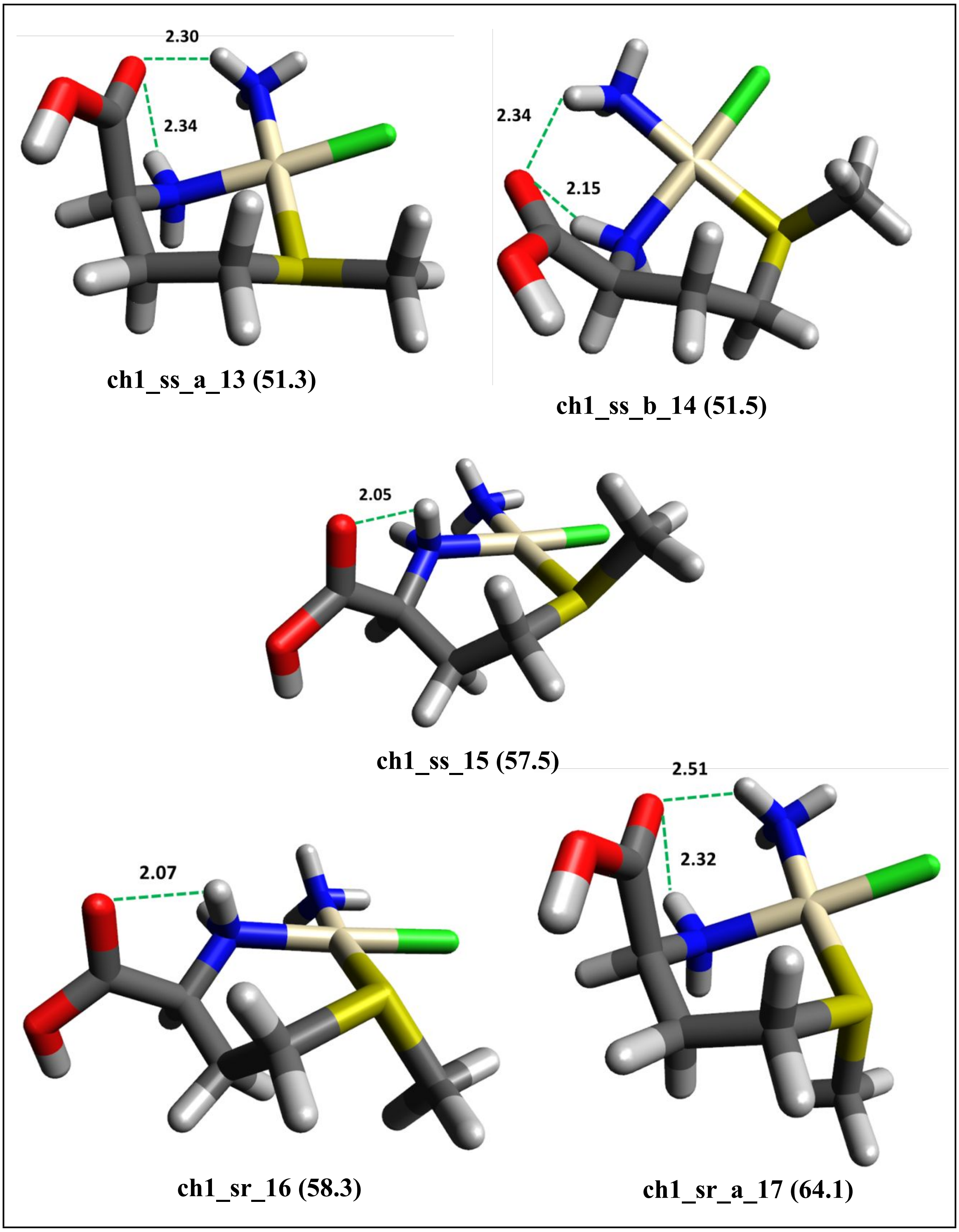

Figure S5 (continuation). Optimized geometries for **ch1** and **ch2** isomers, $[PtCl(NH_3)Met]^+$. Relative Gibbs energy values (kJ mol^{-1}) are reported in parenthesis. Intramolecular hydrogen bond distances (Å) are represented by green dashed lines.

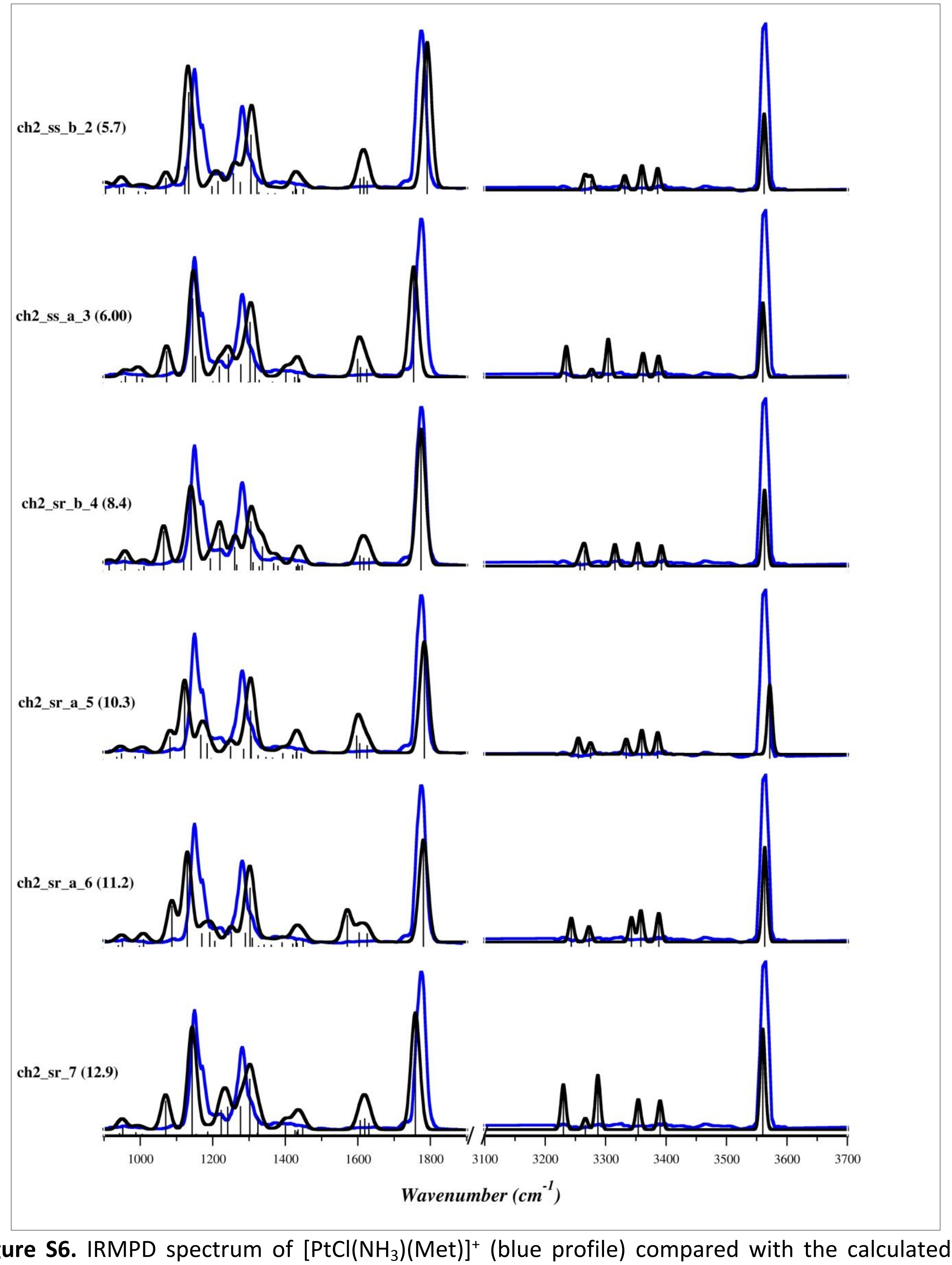

Figure S6. IRMPD spectrum of $[PtCl(NH_3)(Met)]^+$ (blue profile) compared with the calculated IR spectra (black profiles) of the lowest lying conformers of the **ch1** and **ch2** families, computed at B3LYP/BS1 level of theory. Theoretical frequencies have been scaled by 0.974 and 0.957 in the 900-1900 cm^{-1} and the 3100-3700 cm^{-1} ranges, respectively. Free energies relative to **ch2_ss_b_1** are reported in brackets (kJ mol^{-1}).

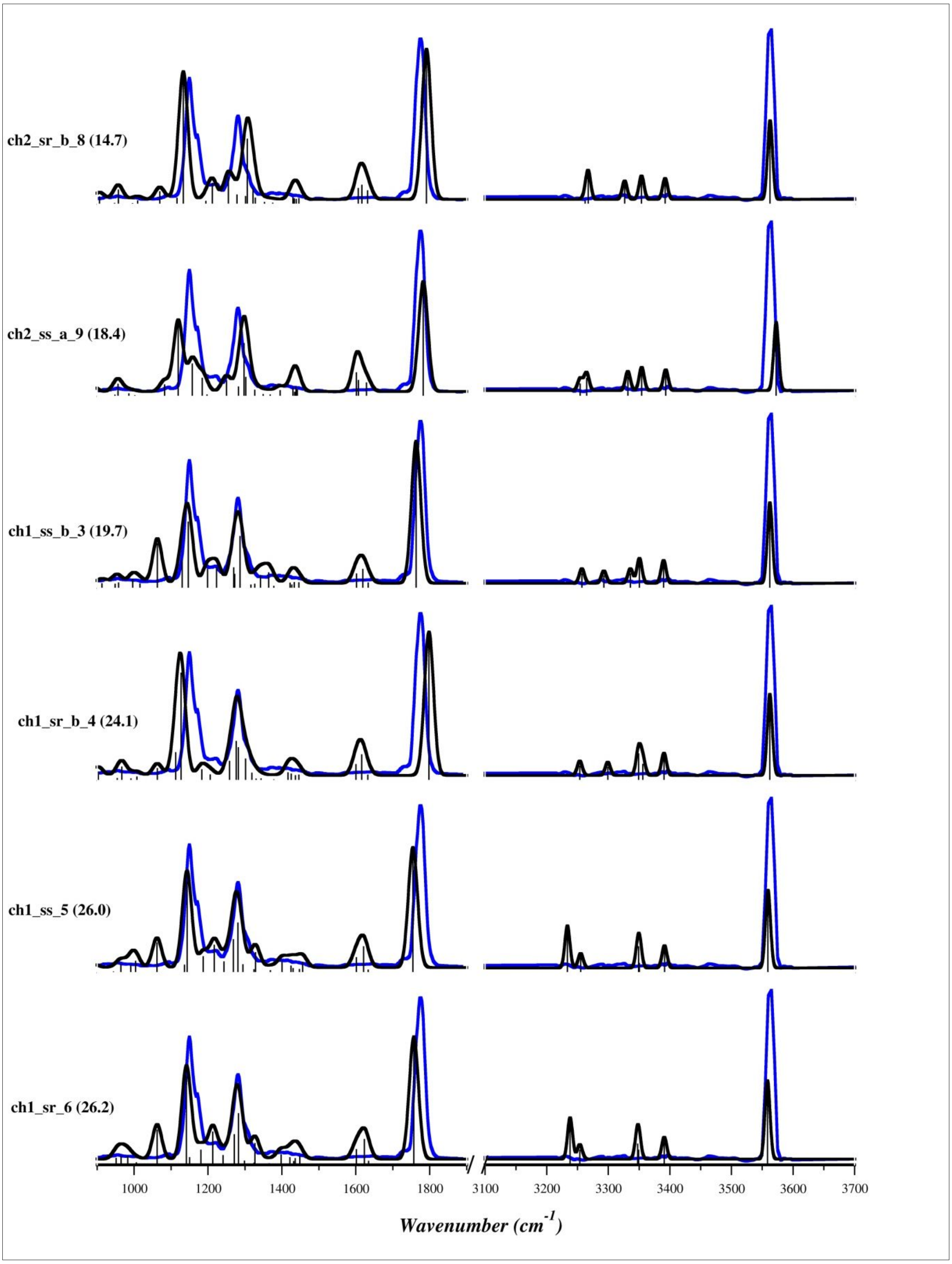

Figure S6 (continuation). IRMPD spectrum of $[PtCl(NH_3)(Met)]^+$ (blue profile) compared with the calculated IR spectra (black profiles) of the lowest lying conformers of the **ch1** and **ch2** families, computed at B3LYP/BS1 level of theory. Theoretical frequencies have been scaled by 0.974 and 0.957 in the 900-1900 cm^{-1} and the 3100-3700 cm^{-1} ranges, respectively. Free energies relative to **ch2_ss_b_1** are reported in brackets (kJ mol^{-1}).

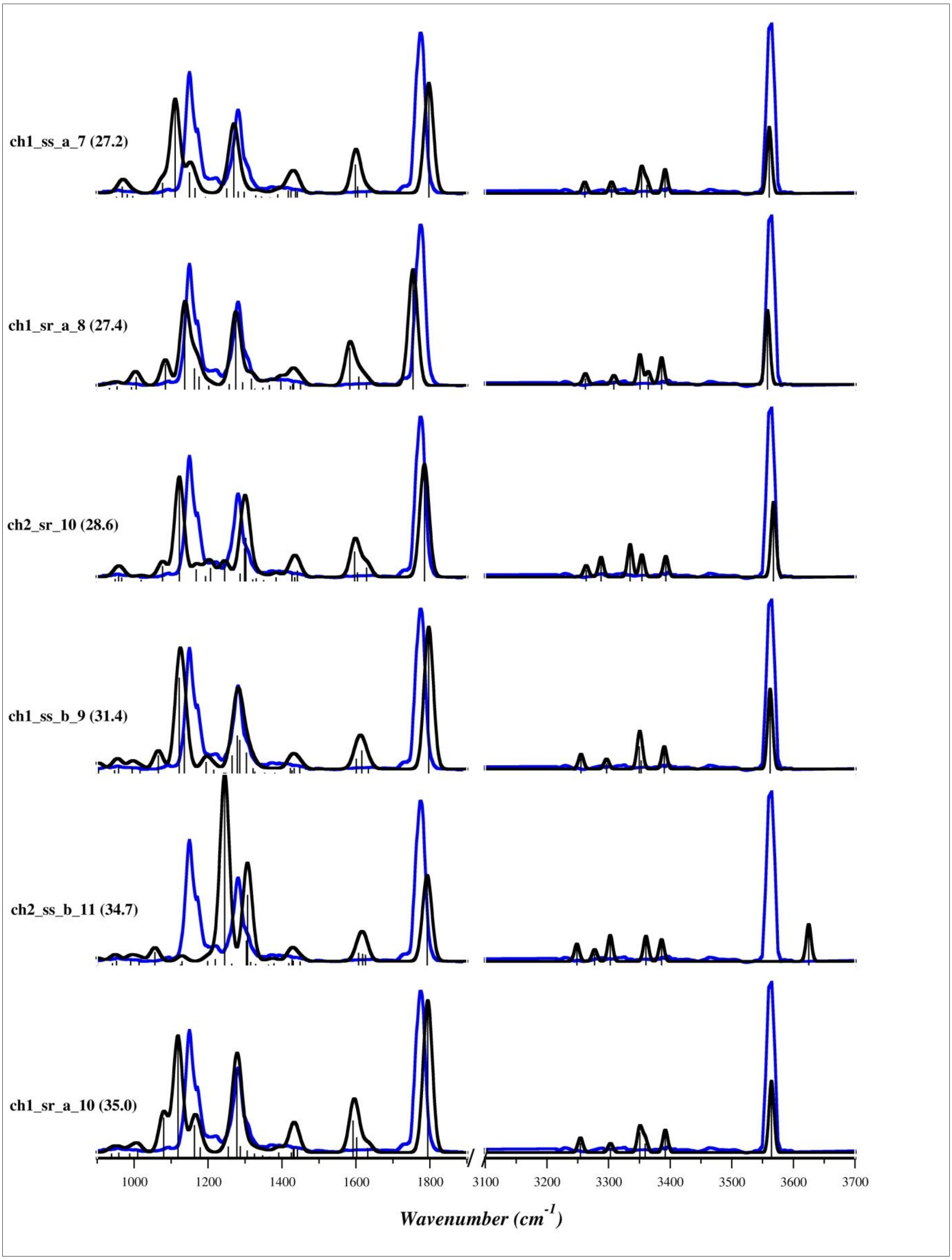

Figure S6 (continuation). IRMPD spectrum of $[PtCl(NH_3)(Met)]^+$ (blue profile) compared with the calculated IR spectra (black profiles) of the lowest lying conformers of the **ch1** and **ch2** families, computed at B3LYP/BS1 level of theory. Theoretical frequencies have been scaled by 0.974 and 0.957 in the 900-1900 cm^{-1} and the 3100-3700 cm^{-1} ranges, respectively. Free energies relative to **ch2_ss_b_1** are reported in brackets (kJ mol^{-1}).

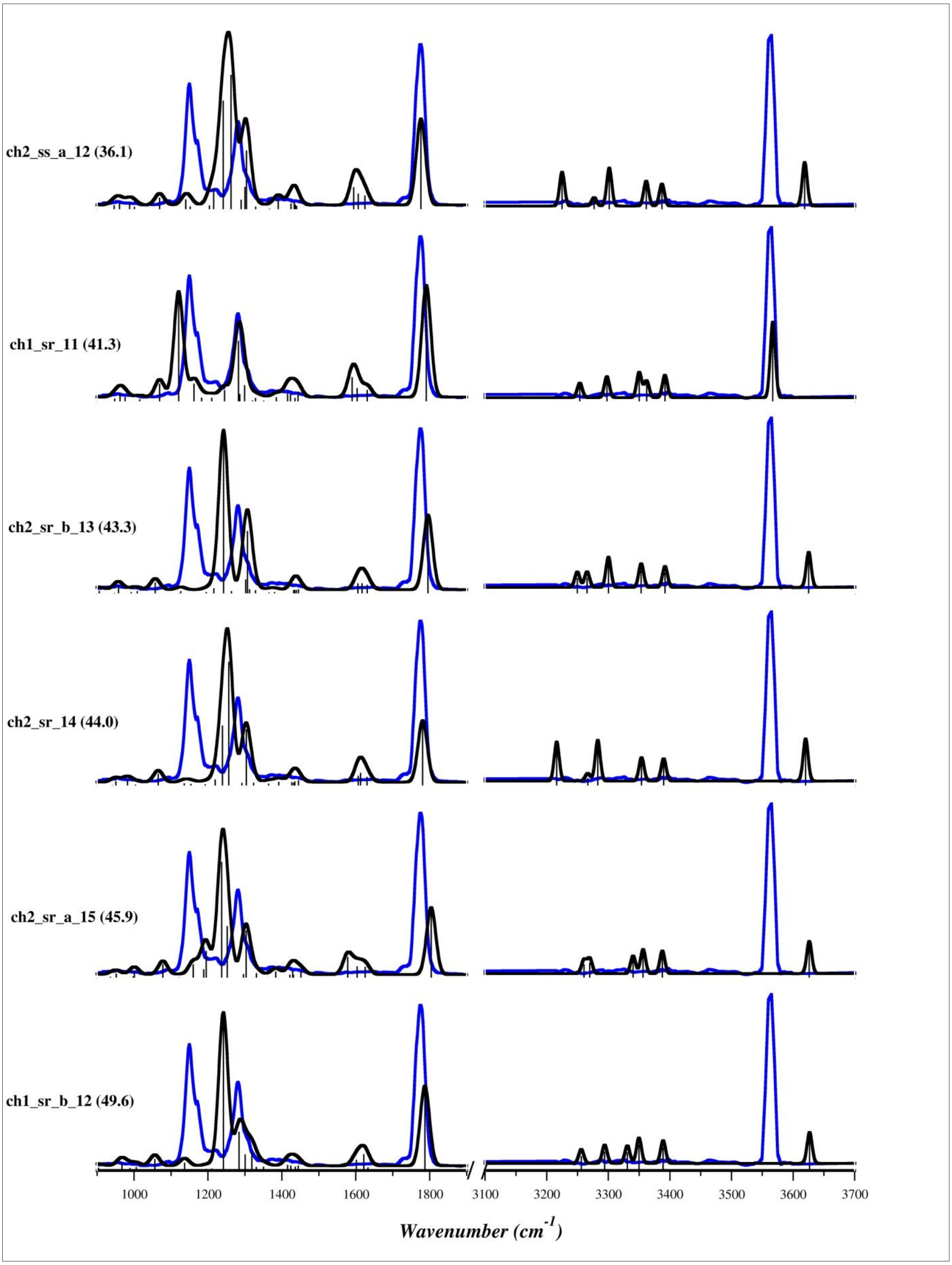

Figure S6 (continuation). IRMPD spectrum of $[PtCl(NH_3)(Met)]^+$ (blue profile) compared with the calculated IR spectra (black profiles) of the lowest lying conformers of the **ch1** and **ch2** families, computed at B3LYP/BS1 level of theory. Theoretical frequencies have been scaled by 0.974 and 0.957 in the 900-1900 cm^{-1} and the 3100-3700 cm^{-1} ranges, respectively. Free energies relative to **ch2_ss_b_1** are reported in brackets (kJ mol^{-1}).

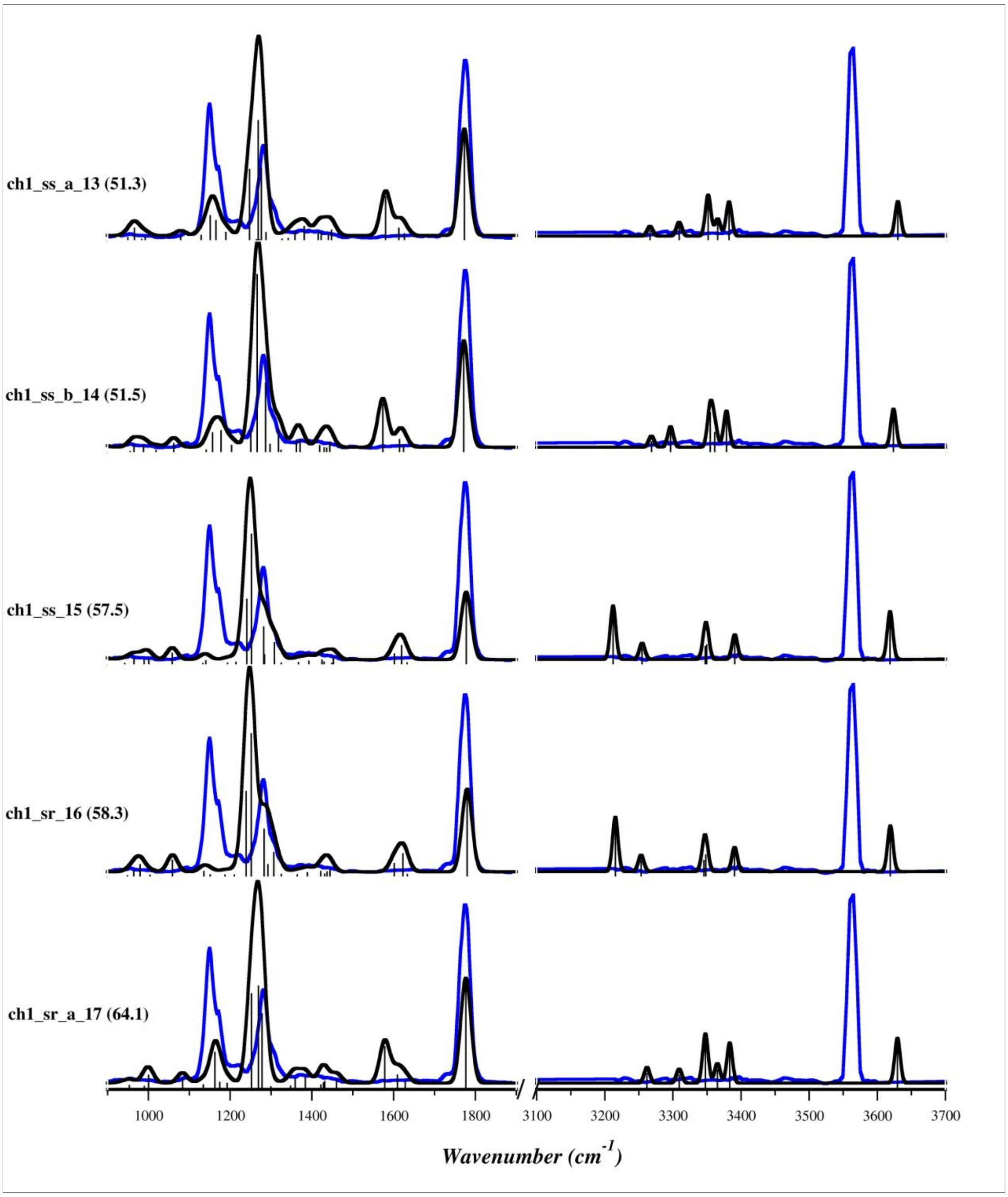

Figure S6 (continuation). IRMPD spectrum of $[PtCl(NH_3)(Met)]^+$ (blue profile) compared with the calculated IR spectra (black profiles) of the lowest lying conformers of the **ch1** and **ch2** families, computed at B3LYP/BS1 level of theory. Theoretical frequencies have been scaled by 0.974 and 0.957 in the 900-1900 cm^{-1} and the 3100-3700 cm^{-1} ranges, respectively. Free energies relative to **ch2_ss_b_1** are reported in brackets (kJ mol^{-1}).

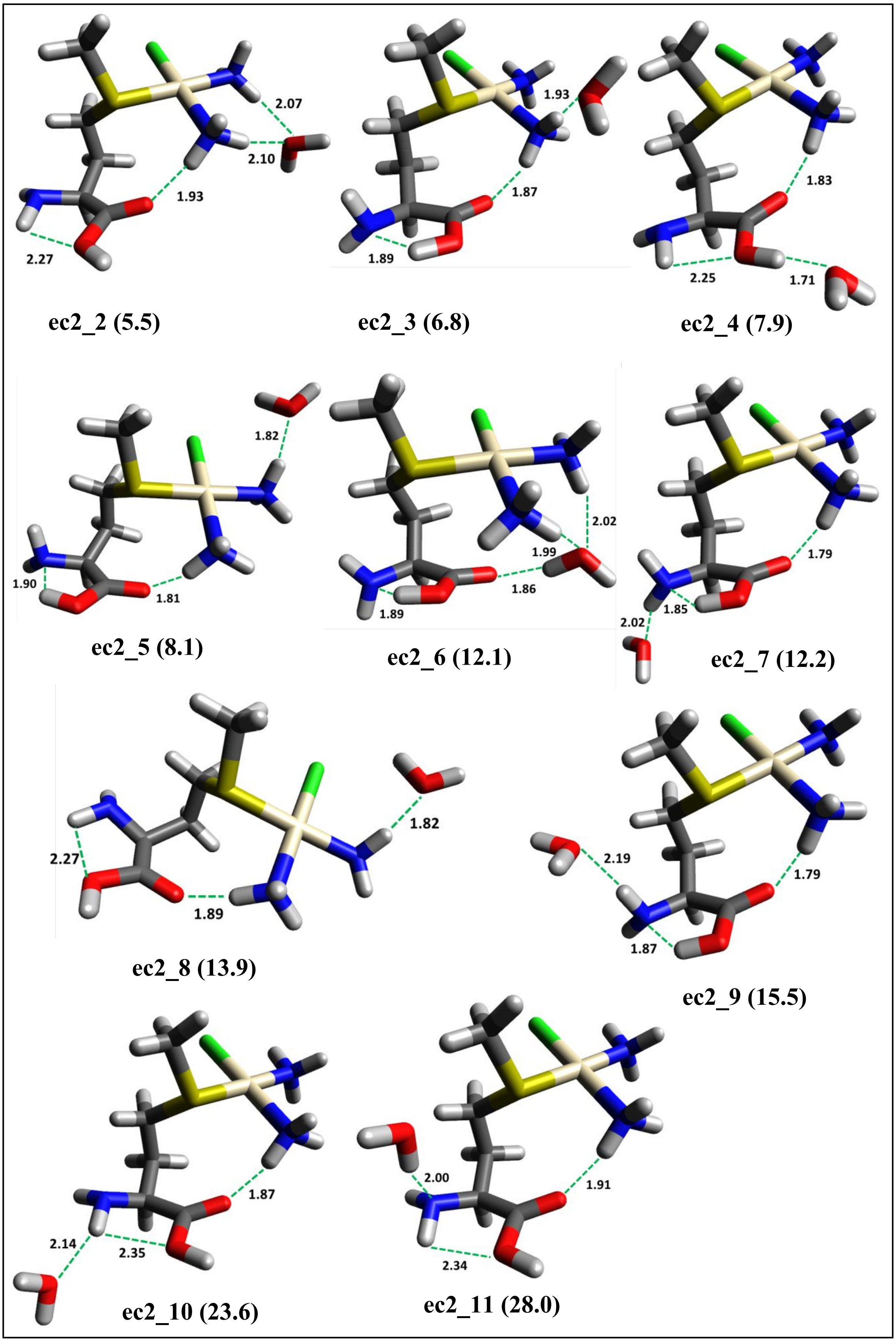

Figure S7. Optimized geometries for **ec2** isomers, {cis-[PtCl(NH_3)$_2$Met]$^+$ • H_2O}. Relative Gibbs energy values (kJ mol^{-1}) are reported in parenthesis. Hydrogen bond distances (Å) are indicated by green dashed lines.

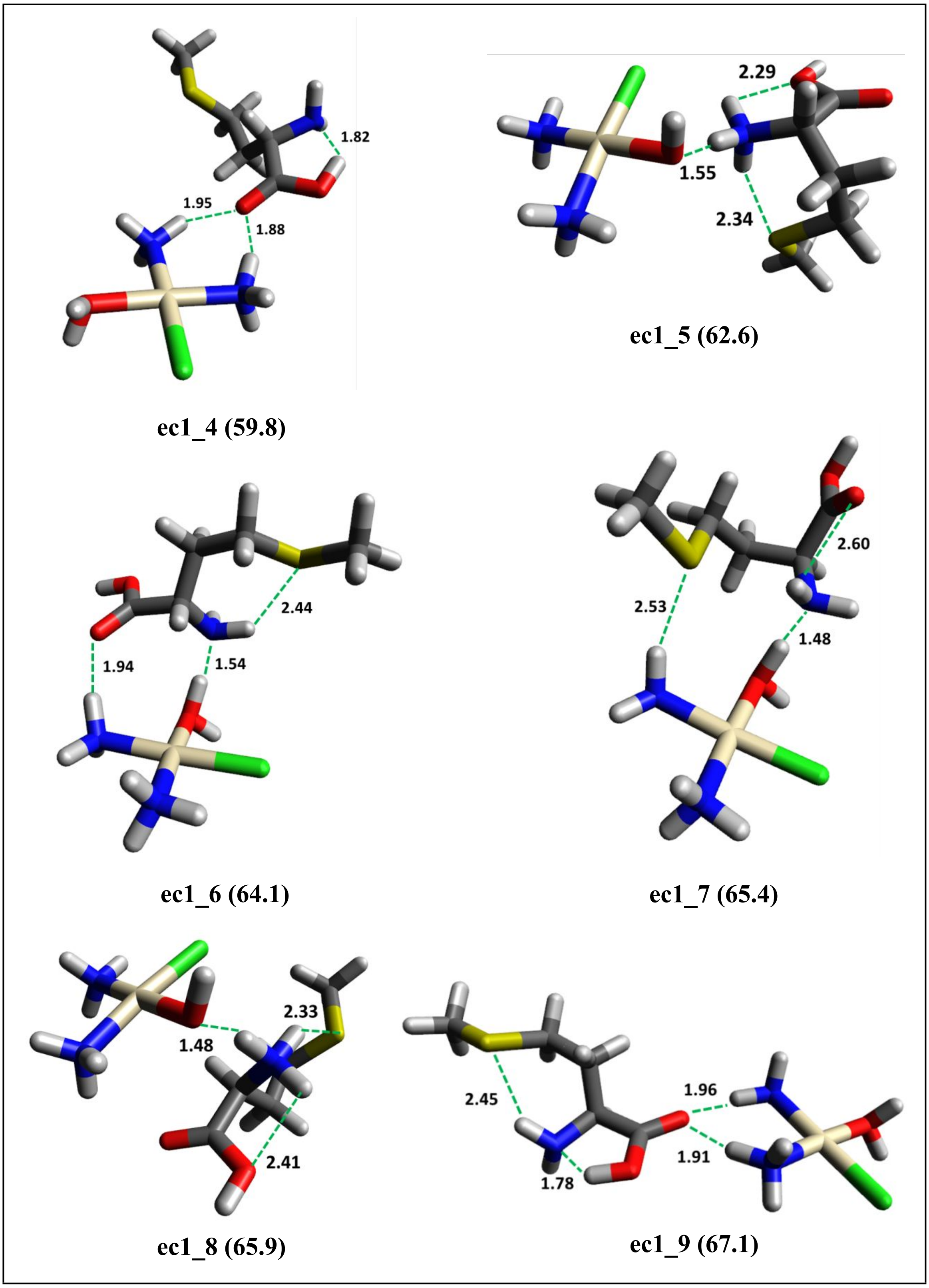

Figure S8. Optimized geometries for **ec1** isomers, {cis-[PtCl(NH_3)$_2$(H_2O)]$^+$ • Met} and {cis-[PtCl(NH_3)$_2$(OH)]• MetH$^+$}. Relative Gibbs energy values (kJ mol^{-1}) are reported in parenthesis. Hydrogen bond distances (Å) are indicated by green dashed lines.

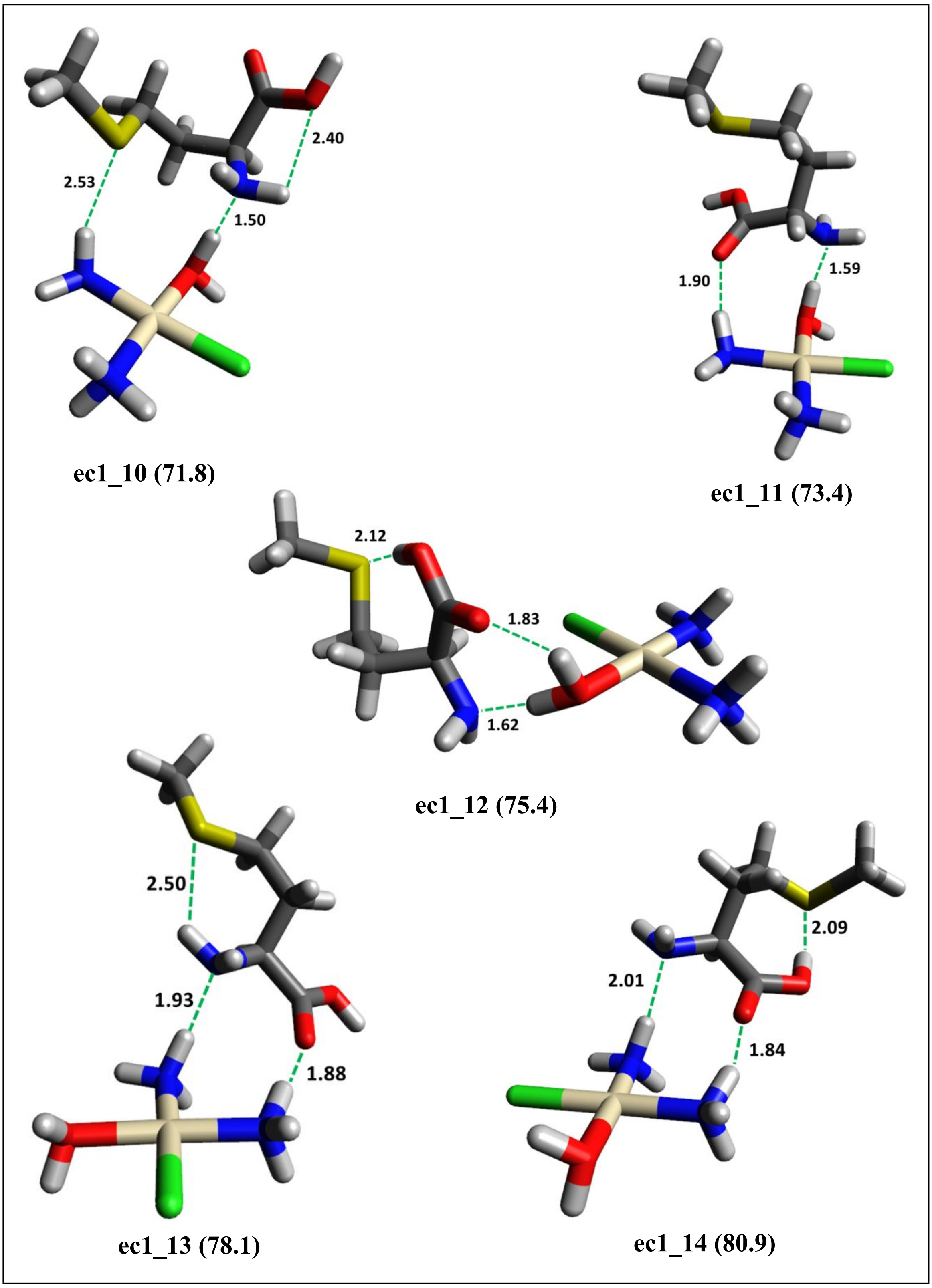

Figure S8 (continuation). Optimized geometries for **ec1** isomers, {cis-[PtCl$(NH_3)_2(H_2O)]^+$ • Met}. Relative Gibbs energy values (kJ mol^{-1}) are reported in parenthesis. Hydrogen bond distances (Å) are indicated by green dashed lines.

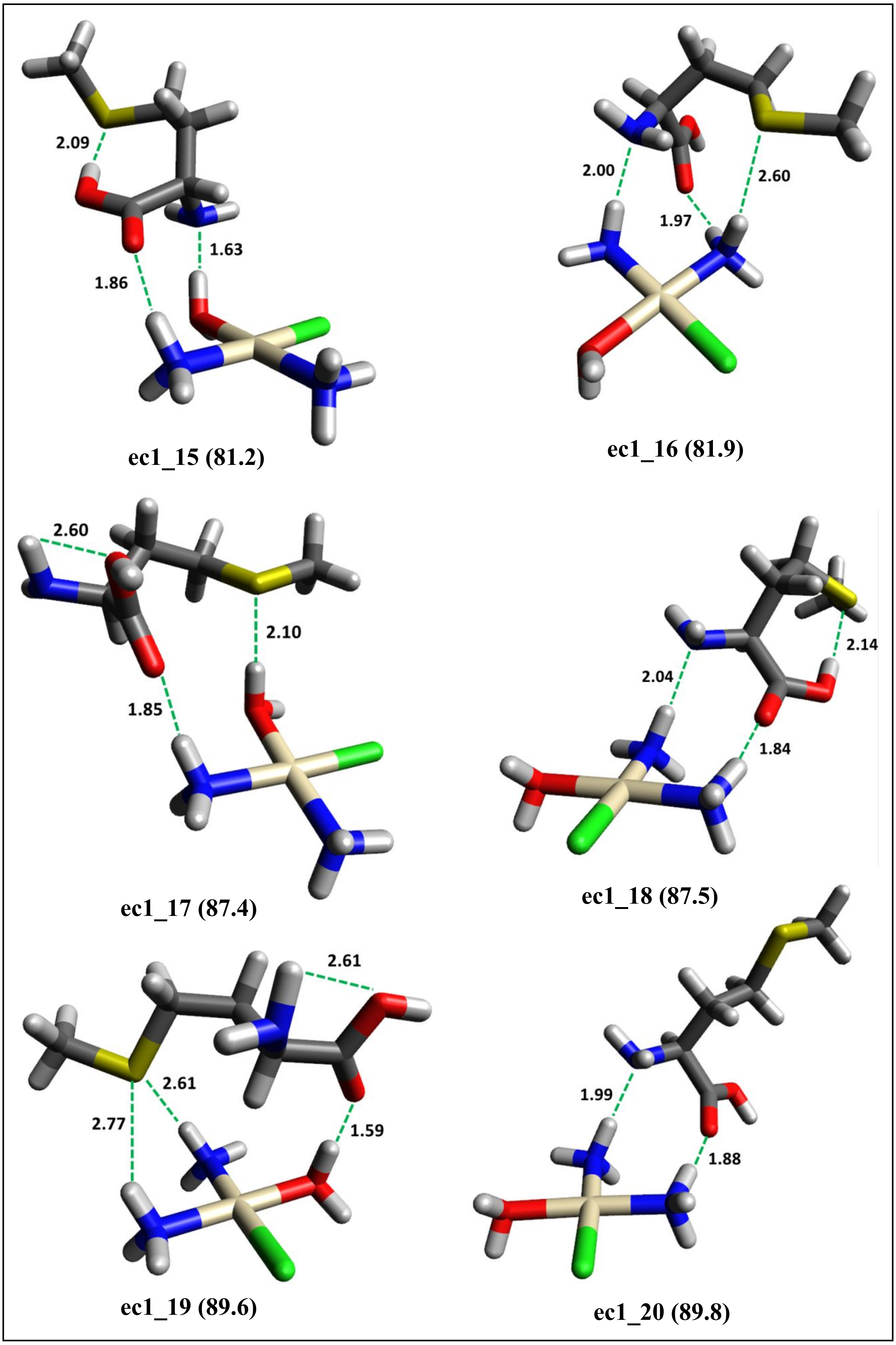

Figure S8 (continuation). Optimized geometries for **ec1** isomers, {cis-[PtCl$(NH_3)_2(H_2O)$]$^+$ • Met}. Relative Gibbs energy values (kJ mol^{-1}) are reported in parenthesis. Hydrogen bond distances (Å) are indicated by green dashed lines.

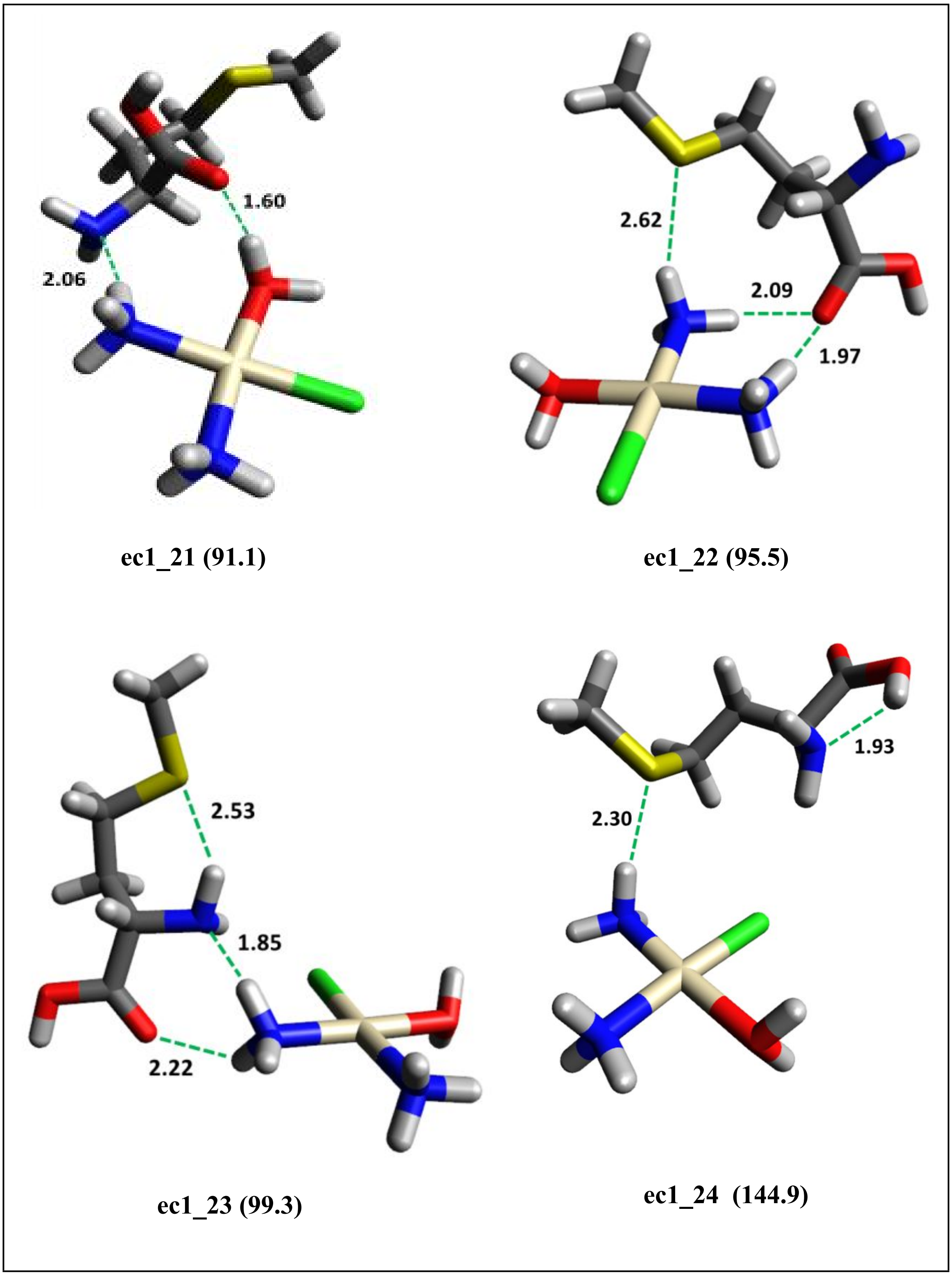

Figure S8 (continuation). Optimized geometries for **ec1** isomers, {cis-[PtCl$(NH_3)_2(H_2O)]^+$ • Met}. Relative Gibbs energy values (kJ mol^{-1}) are reported in parenthesis. Hydrogen bond distances (Å) are indicated by green dashed lines.

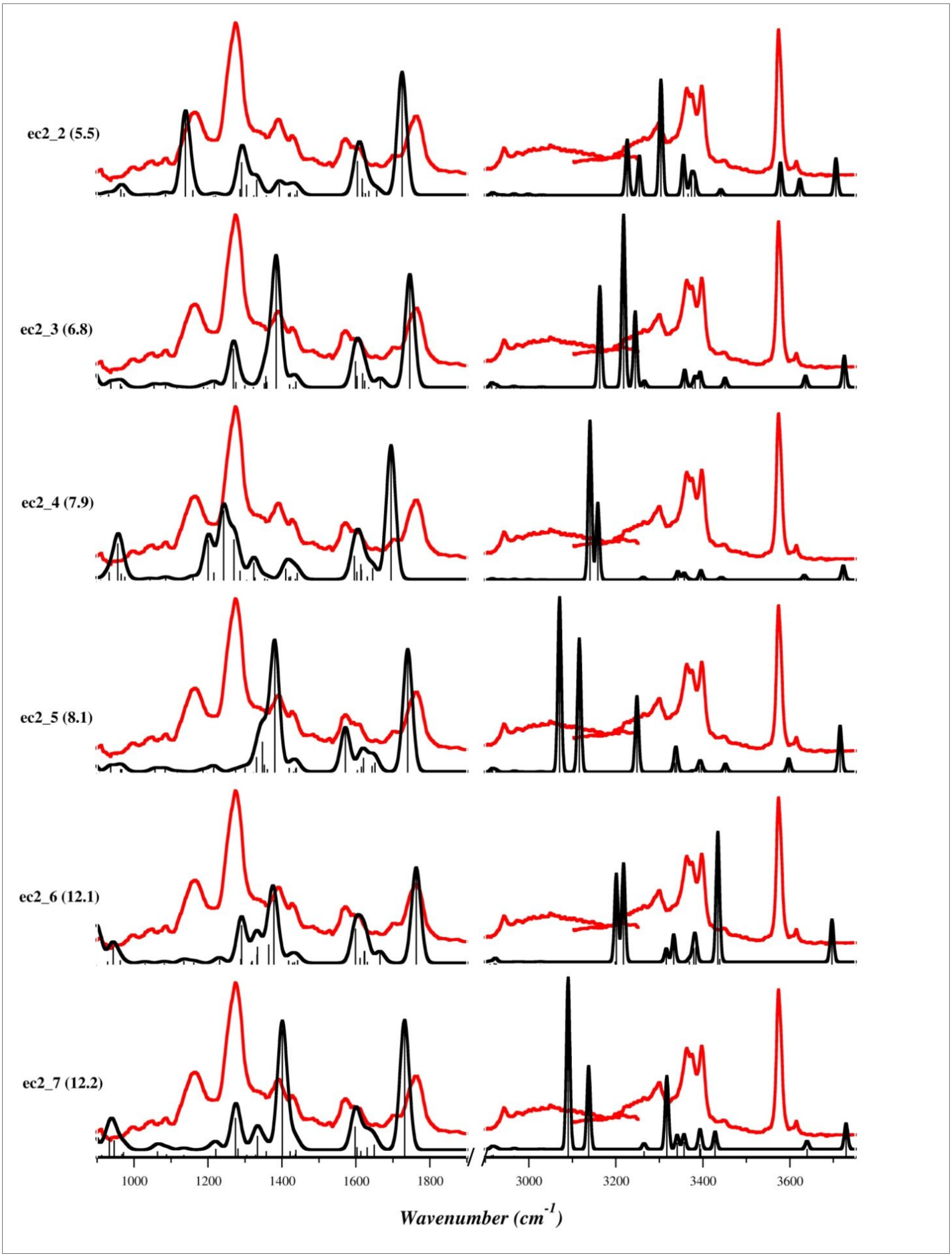

Figure S9. IRMPD spectrum of $[PtCl(NH_3)_2(H_2O)(Met)]^+$ (red profile) compared with the calculated IR spectra (black profiles) of the lowest lying geometries of the **ec2** and **ec1** isomer families, computed at B3LYP/BS1 level of theory. Theoretical frequencies have been scaled by 0.974 and 0.957 in the 900-1900 cm^{-1} and the 3000-3700 cm^{-1} ranges, respectively. Free energies relative to **ec2_1** are reported in brackets (kJ mol^{-1}).

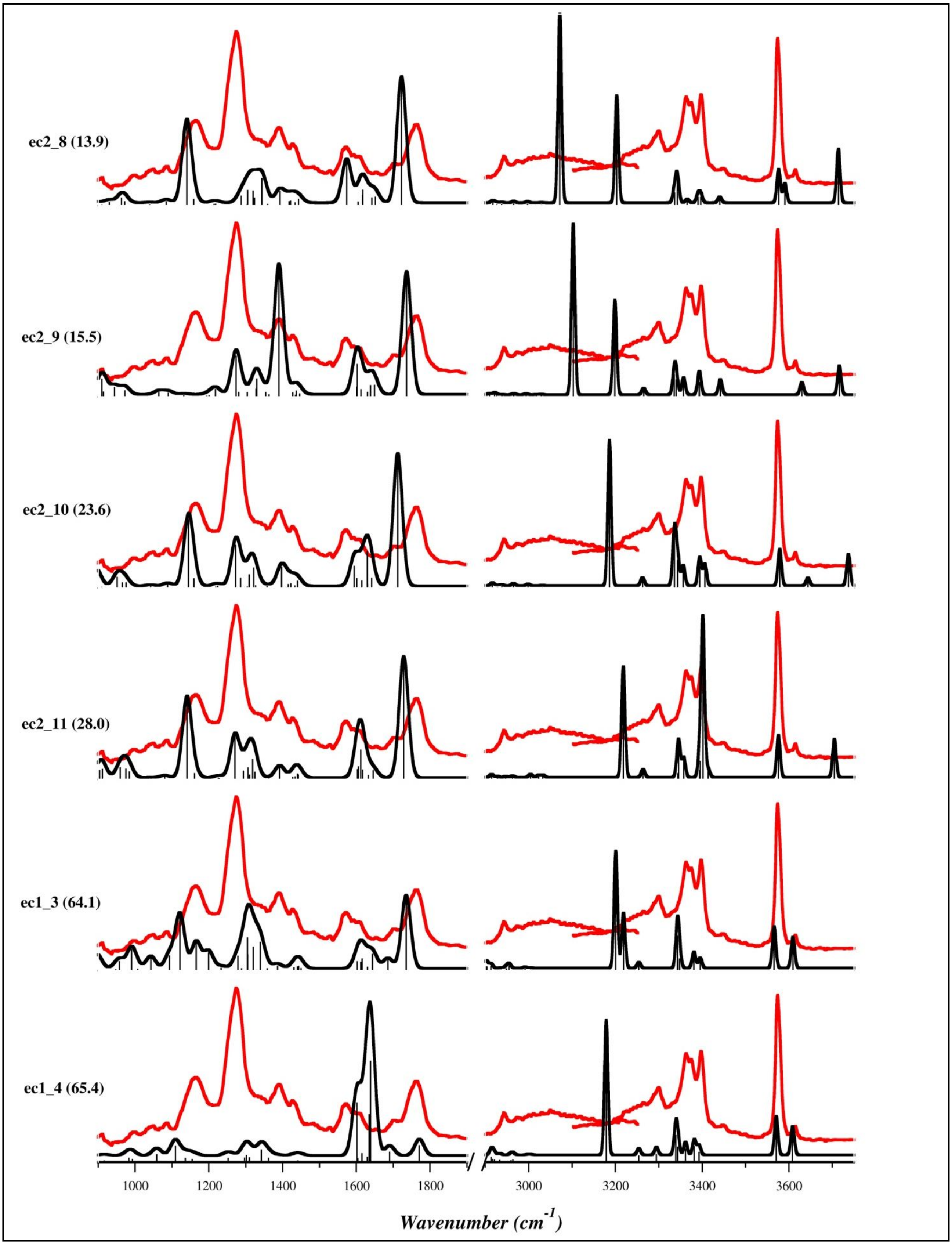

Figure S9 (continuation). IRMPD spectrum of $[PtCl(NH_3)_2(H_2O)(Met)]^+$ (red profile) compared with the calculated IR spectra (black profiles) of the lowest lying geometries of the **ec2** and **ec1** isomer families, computed at B3LYP/BS1 level of theory. Theoretical frequencies have been scaled by 0.974 and 0.957 in the 900-1900 cm^{-1} and the 3000-3700 cm^{-1} ranges, respectively. Free energies relative to **ec2_1** are reported in brackets (kJ mol^{-1}).

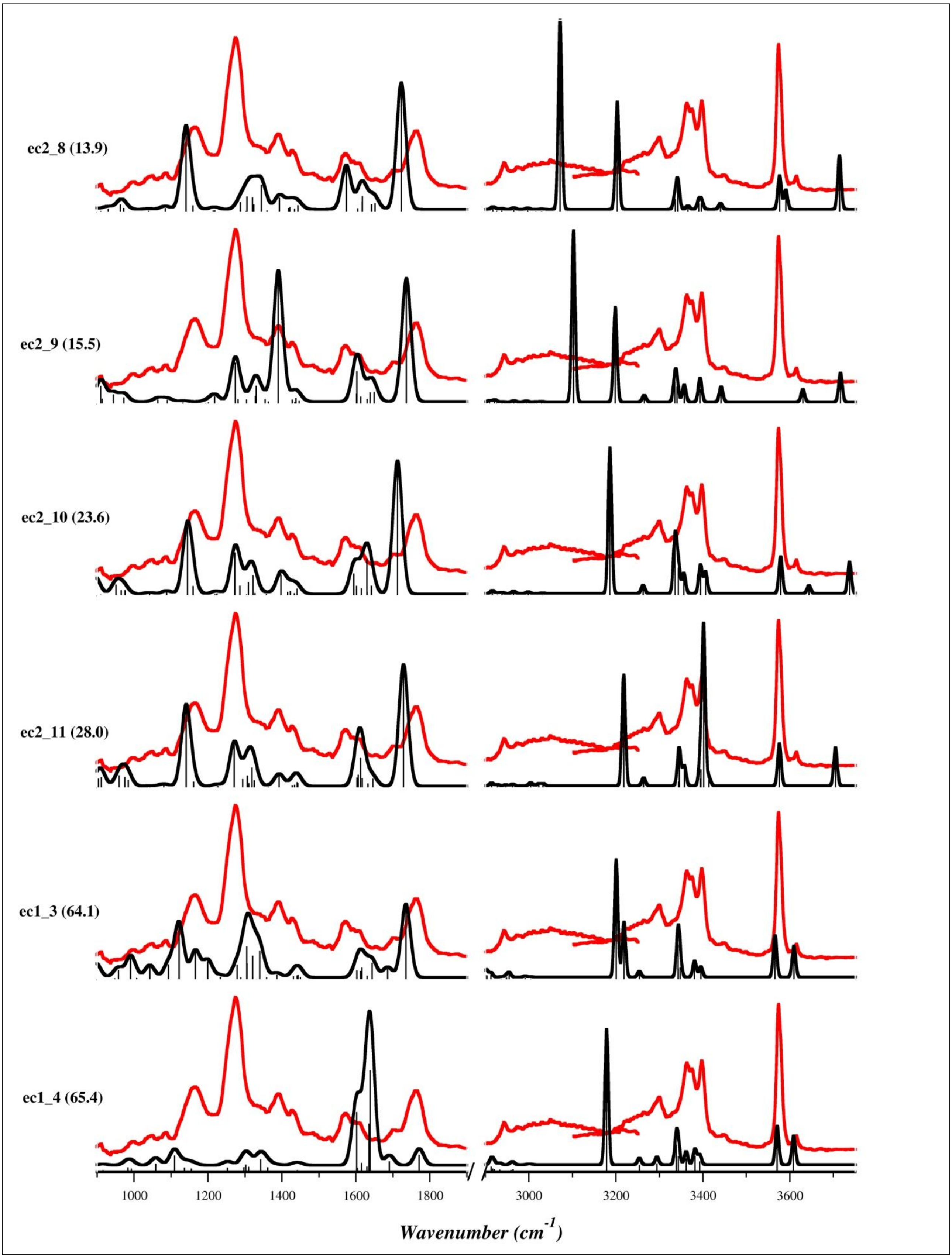

Figure S9 (continuation). IRMPD spectrum of $[PtCl(NH_3)_2(H_2O)(Met)]^+$ (red profile) compared with the calculated IR spectra (black profiles) of the lowest lying geometries of the **ec2** and **ec1** isomer families, computed at B3LYP/BS1 level of theory. Theoretical frequencies have been scaled by 0.974 and 0.957 in the 900-1900 cm^{-1} and the 3000-3700 cm^{-1} ranges, respectively. Free energies relative to **ec2_1** are reported in brackets (kJ mol^{-1}).

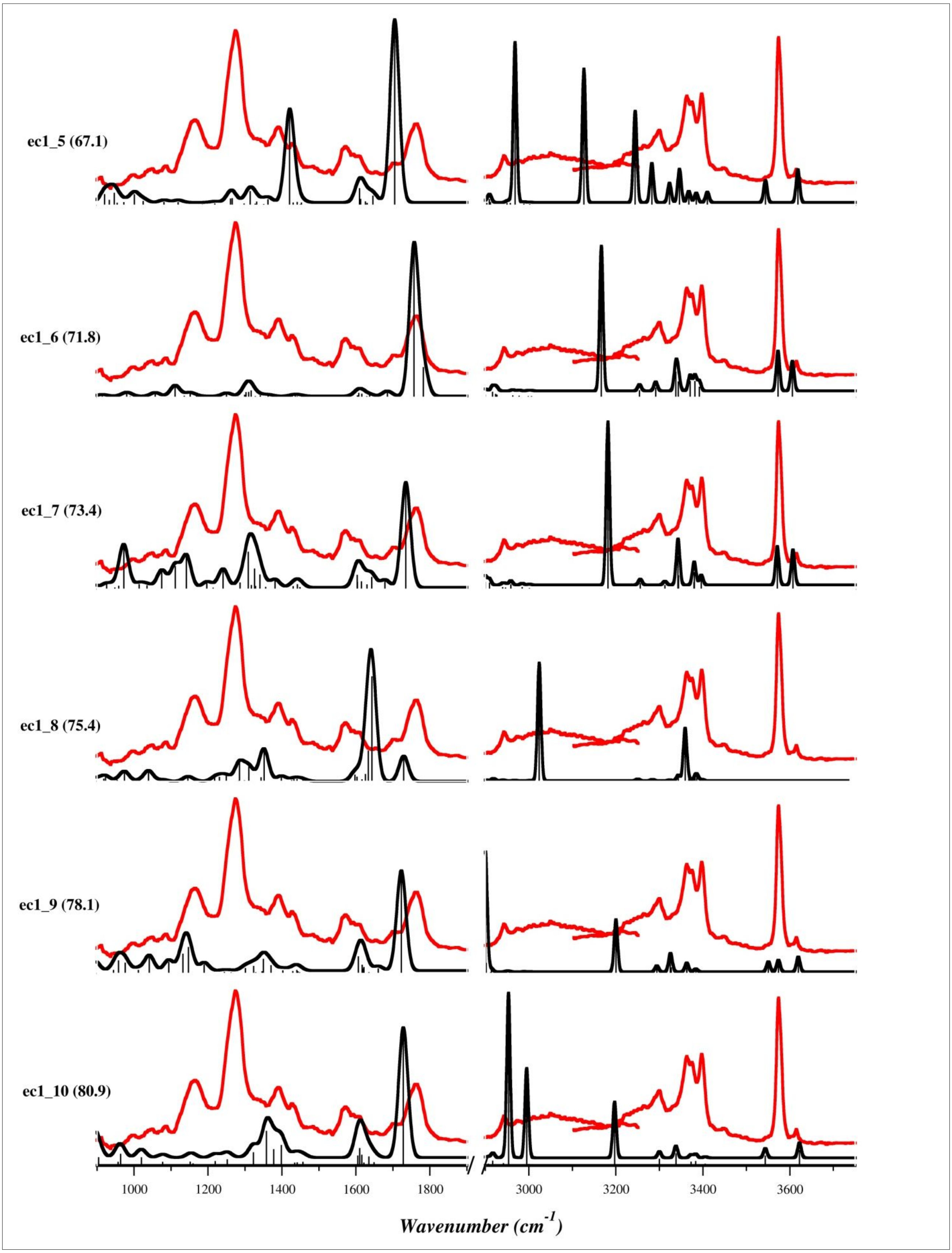

Figure S9 (continuation). IRMPD spectrum of $[PtCl(NH_3)_2(H_2O)(Met)]^+$ (red profile) compared with the calculated IR spectra (black profiles) of the lowest lying geometries of the **ec2** and **ec1** isomer families, computed at B3LYP/BS1 level of theory. Theoretical frequencies have been scaled by 0.974 and 0.957 in the 900-1900 cm^{-1} and the 3000-3700 cm^{-1} ranges, respectively. Free energies relative to **ec2_1** are reported in brackets (kJ mol^{-1}).

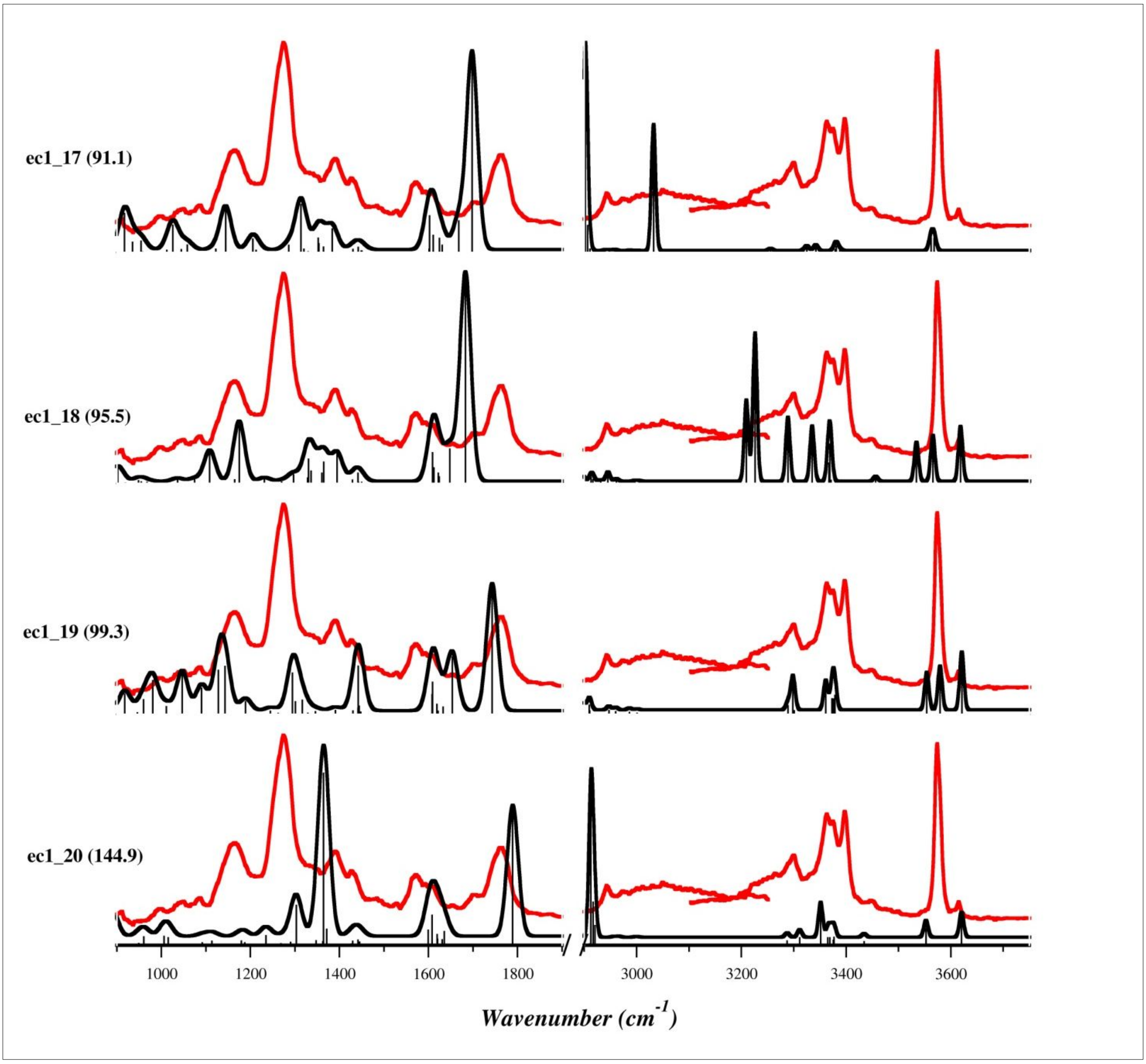

Figure S9 (continuation). IRMPD spectrum of $[PtCl(NH_3)_2(H_2O)(Met)]^+$ (red profile) compared with the calculated IR spectra (black profiles) of the lowest lying geometries of the **ec2** and **ec1** isomer families, computed at B3LYP/BS1 level of theory. Theoretical frequencies have been scaled by 0.974 and 0.957 in the 900-1900 cm^{-1} and the 3000-3700 cm^{-1} ranges, respectively. Free energies relative to **ec2_1** are reported in brackets (kJ mol^{-1}).

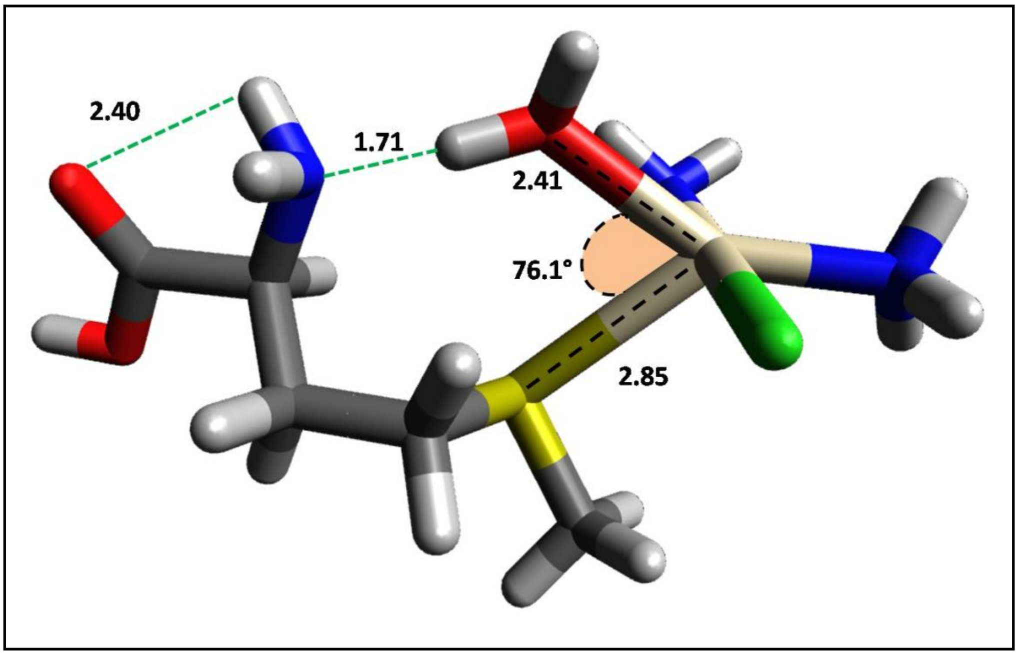

Figure S10: Structure of transition state participating in the reaction pathway for the ligand substitution reaction of cis-[PtCl$(NH_3)_2(H_2O)$]$^+$ with Met. Bond lengths are in Å. The structural motives characterizing the only negative vibrational frequency are highlighted by the black dashed lines while H bonds are reported as green dashed lines.

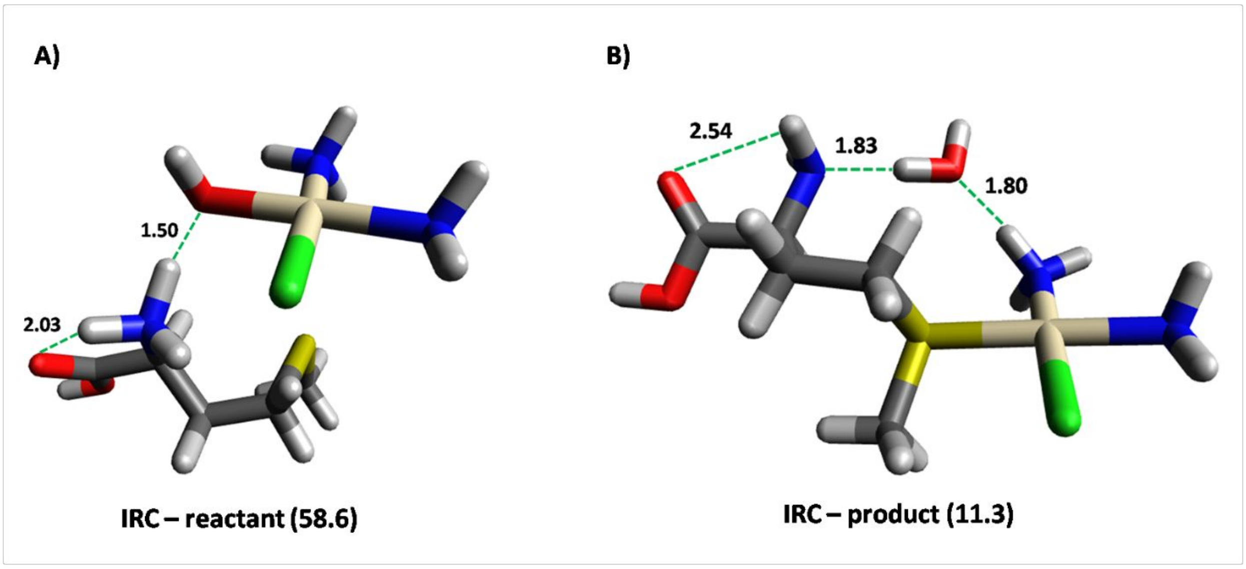

Figure S11: Optimized geometries of the reactant (A) and of the product (B) obtained by intrinsic reaction coordinate (IRC) calculations. Free energy values at 298 K (kJ mol^{-1}) relative to **ec_2-1** are shown in brackets. The hydrogen bond (Å) lengths are indicated by green dashed lines.

Table S1. IRMPD absorptions of $[PtCl(NH_3)(Met)]^+$ ions and calculated vibrational frequencies (cm^{-1}) for **ch1_sr_b_1** and **ch1_ss_a_2**. Calculated intensities are reported in brackets (km mol^{-1}). Modes with intensity lower than 15 km mol^{-1} are omitted.

Calculated[#]		Exp	Vibrational mode
**ch1_sr_b_1**	**ch1_ss_a_2**	$[PtCl(NH_3)(Met)]^+$	
967[a] (28)		960	CH_3 asymm bending
1062[a] (77)	1080[a] (33)	1090	CN stretching + CC stretching + OH bending
1150[a] (178)	1129[a] (133)	1148	OH bending + CH_2 scissoring
	1161[a] (56)	1175 (shoulder)	OH bending + NH_2 wagging+ CH_2 twisting
	1163[a] (63)		OH bending + NH_2 wagging
1190[a] (36)			NH_2 wagging + CH_2 twisting+ CH bending
1210[a] (56)		1222	NH_2 wagging + CH_2 twisting
1260[a] (30)			CH_2 wagging + NH_2 twisting
1271[a] (34)			CH bending + OH bending
1282[a] (111)	1273[a] (142)	1280	NH_3 umbrella mode
1360[a] (52)		1390	CH bending + C-OH bending
1601[a] (32)		1620	NH_3 asymm bending
1621[a](51)	1585[a] (91)		NH_2 scissoring + NH_3 asymm bending
1760[a] (294)	1750[a] (245)	1777	C=O stretching
3256[b] (28)			NH_3 symm stretching
3287[b] (42)	3309[b] (25)		NH_2 symm stretching
3326[b] (38)			NH_2 asymm stretching
3351[b] (52)	3353[b] (70)		NH_3 asymm stretching
	3365[b] (31)		NH_2 asymm stretching

3390[b] (47)	3383[b] (72)		NH_3 asymm stretching
3560[b] (167)	3558[b] (151)	3563	OH stretching

[#]Theoretical IR frequencies were computed at B3LYP/BS1 level of theory.

[a] Frequencies are scaled by a factor of 0.974.

[b] Frequencies are scaled by a factor of 0.957.

Table S2. IRMPD absorptions of $[PtCl(NH_3)_2(H_2O)(Met)]^+$ ions and calculated vibrational frequencies (cm^{-1}) for **ec1_1** and **ec1_2**. Calculated intensities are reported in brackets (km mol^{-1}). Modes with intensity lower than 15 km mol^{-1} are omitted.

Calculated[#]		Experimental	Vibrational mode
**ec1_1**	**ec1_2**	$[PtCl(NH_3)_2(H_2O)(Met)]^+$	
990[a] (173)		1000	NH_2 wagging + CH_2 rocking
1049[a] (268)		1044	CN stretch + H_2O wagging
	1072[a] (36)	1086	CN stretch + CH_2 rocking
1146[a] (232)		1166	C(O)O-H bending
	1124[a] (115)		C(O)O-H bending + CH_2 twisting + NH_3asymm bending
	1146[a] (72)		C(O)O-H bending + CH_2 wagging + NH_3asymm bending
	1155[a] (34)		C(O)O-H bending + CH_2 twisting + NH_3asymm bending
	1172[a] (54)		C(O)O-H bending + NH_3asymm bending
1185[a] (118)			NH_2 twisting + CH bending
1222[a] (1217)		1273	CH bending + HO-H stretching + C(O)O-H bending + CH_2 twisting
1258[a] (783)			CH bending + HO-H stretching
	1263[a] (119)		NH_3 (trans Cl) umbrella
1271[a] (153)			CH bending
1289[a] (380)			NH_3 (trans Cl) umbrella
	1296[a] (122)		NH_3 umbrella
1305[a] (233)			NH_3 umbrella + NH_2 twisting + CH_2 twisting
1306[a] (230)			NH_3 umbrella
1343[a] (629)		1390	C(O)O-H bending + CH bending + C=O stretching + HO-H stretching
1352[a] (92)			CH_2 wagging
	1391[a] (21)		CH bending
1397[a] (320)		1426	CH bending + HO-H bending
1562[a] (69)		1573	NH_3 (methionine) umbrella
1606[a] (41)			NH_2 bending
1619[a] (44)	1606[a] (29)	1611	NH_3 bending

1635[a] (52)	1627[a] (34)		NH_3 bending
	1636[a] (41)		NH_3 bending
	1641[a] (108)		NH_3 (methionine) bending
1707[a] (110)		1708	H_2O scissoring + C=O stretching
1764[a] (419)	1768[a] (238)	1766	C=O stretching + H_2O scissoring,
2906[b] (21)	2916[b] (19)	2943	CH_2 asymm stretching
	3084[b] (504)	3050 (broad)	NH of NH_3 (methionine) stretching
3255[b] (144)		3250 (broad)	NH_2 symm stretching
3282[b] (15)			NH_3 symm stretching
	3301[b] (78)	3300	NH of NH_3 (methionine) stretching
3346[b] (54)		3363	NH_3 asymm stretching
3348[b] (43)			NH_2 asymm stretching
3367[b] (54)	3353[b] (45)	3375	NH_3 asymm stretching
	3360[b] (58)		NH_3 asymm stretching
3383[b] (31)	3387[b] (33)	3400	NH_3 asymm stretching
3388[b] (60)	3391[b] (51)		NH_3 asymm stretching
3552[b] (201)		3574	HO-H stretching
3575[b] (115)	3571[b] (137)		C(O)O-H stretching
	3636[b] (41)	3615	PtO-H stretching

[#]Theoretical IR frequencies were computed at B3LYP/BS1 level of theory.
[a] Frequencies are scaled by a factor of 0.974.
[b] Frequencies are scaled by a factor of 0.957.

Table S3: Cartesian coordinates of transition state computed for the aqua ligand substitution reaction of cis-[PtCl$(NH_3)_2(H_2O)]^+$ with Met

H	2.994278	-0.342018	-1.015799
C	3.387284	-0.247360	0.002416
C	4.895141	-0.476204	-0.108112
O	5.475580	-1.390714	0.412460
C	3.066246	1.164105	0.512222
N	2.755195	-1.284033	0.831008
H	3.493160	1.884168	-0.185441
H	3.567552	1.321181	1.474656
O	5.485780	0.447526	-0.882250
H	6.433659	0.242693	-0.935604
Pt	-1.722375	-0.313712	-0.100216
O	0.124782	-1.790971	0.338524
N	-3.755949	0.027694	-0.128590
H	-4.057994	0.644132	-0.879567
H	-4.316884	-0.820721	-0.163959
H	-3.954841	0.493936	0.760631
N	-1.576121	-1.337932	-1.925618
H	-1.258090	-0.747589	-2.691496
H	-0.851900	-2.037951	-1.755668
H	-2.406807	-1.828895	-2.246559
Cl	-1.781714	0.691345	1.966179
C	1.578960	1.443944	0.717869
H	1.441224	2.475794	1.037298
H	1.143790	0.811661	1.489124
S	0.573624	1.228254	-0.806939
C	-0.178523	2.874556	-0.962496
H	-0.785396	3.097460	-0.087145
H	-0.806675	2.857367	-1.851850
H	0.600180	3.624001	-1.095011
H	1.086917	-1.543988	0.543978
H	-0.178357	-2.312907	1.091219
H	2.952699	-1.101108	1.813335
H	3.214221	-2.173660	0.641856
